# The heat shock factor family from *Triticum aestivum* in response to heat and other major abiotic stresses and their role in regulation of heat shock protein genes

**DOI:** 10.1093/jxb/ert399

**Published:** 2013-12-09

**Authors:** Gang-Ping Xue, Shahab Sadat, Janneke Drenth, C. Lynne McIntyre

**Affiliations:** ^1^CSIRO Plant Industry, 306 Carmody Rd, St Lucia, Qld 4067, Australia; ^2^Department of Plant Breeding, Science and Research Branch, Islamic Azad University, Tehran, Iran

**Keywords:** Gene expression, gene regulation, heat shock factors, heat shock proteins, heat stress, transcription factors, wheat.

## Abstract

Heat shock factors (Hsfs) play a central regulatory role in acquired thermotolerance. To understand the role of the major molecular players in wheat adaptation to heat stress, the Hsf family was investigated in *Triticum aestivum*. Bioinformatic and phylogenetic analyses identified 56 TaHsf members, which are classified into A, B, and C classes. Many TaHsfs were constitutively expressed. Subclass A6 members were predominantly expressed in the endosperm under non-stress conditions. Upon heat stress, the transcript levels of A2 and A6 members became the dominant Hsfs, suggesting an important regulatory role during heat stress. Many TaHsfA members as well as B1, C1, and C2 members were also up-regulated during drought and salt stresses. The heat-induced expression profiles of many heat shock protein (Hsp) genes were paralleled by those of A2 and A6 members. Transactivation analysis revealed that in addition to TaHsfA members (*A2b* and *A4e*), overexpression of *TaHsfC2a* activated expression of TaHsp promoter-driven reporter genes under non-stress conditions, while *TaHsfB1b* and *TaHsfC1b* did not. Functional heat shock elements (HSEs) interacting with TaHsfA2b were identified in four TaHsp promoters. Promoter mutagenesis analysis demonstrated that an atypical HSE (**GAA**CA**TTT**TG**GAA**) in the *TaHsp17* promoter is functional for heat-inducible expression and transactivation by Hsf proteins. The transactivation of Hsp promoter-driven reporter genes by TaHsfC2a also relied on the presence of HSE. An activation motif in the C-terminal domain of TaHsfC2a was identified by amino residue substitution analysis. These data demonstrate the role of HsfA and HsfC2 in regulation of Hsp genes in wheat.

## Introduction

Temperate cereal crops such as wheat often encounter heat stress during the reproductive stage in warm-climate wheat production regions (Wardlaw and Wrigley, 1994). Heat stress has a significant adverse impact on carbon assimilation and starch synthesis in these environments, which leads to reduction of grain yield as well as grain quality (Wardlaw and Wrigley, 1994; Skylas *et al.*, 2002). At the biochemical level, high temperatures cause denaturation of many heat-labile proteins and an elevated level of harmful reactive oxygen species (ROS) in plant cells (Xue and McIntyre, 2011; Mittler *et al.*, 2012; Grover *et al.*, 2013). The acquisition of thermotolerance in plants relies on acclimatization to permissive high temperatures, during which time a large number of heat protection genes such as those encoding heat shock proteins (Hsps) and ROS scavengers are induced (Kotak *et al.*, 2007; Mittler *et al.*, 2012). In this heat acclimatization process, heat shock factors (Hsfs) play an important role in regulation of this heat-induced transcriptional reprogramming (Kotak *et al.*, 2007).

Hsf proteins are transcription factors, which are present in all eukaryotes. Hsfs are characterized by the presence of a Hsf DNA-binding domain, which is composed of a central helix–turn–helix motif and an adjacent bipartite oligomerization domain, made up of of hydrophobic heptad repeats (HR-A and HR-B) (Scharf *et al.*, 2012). The oligomerization domain is required for the formation of a trimeric Hsf structure for high affinity binding to DNA (Peteranderl and Nelson, 1992; Peteranderl *et al.*, 1999). Hsfs are known to recognize the multiple inverted repeats of the nGAAn sequence, known as the heat shock element (HSE), which appears to be present in the promoters of many heat-inducible genes (Santoro *et al.*, 1998; Nover *et al.*, 2001; Guo *et al.*, 2008; Mittal *et al.*, 2011). In general, at least three nGAAn repeats are required for effective Hsf binding in *Drosophila* (Xiao *et al.*, 1991) and in *Arabidopsis* (Kumar *et al.*, 2009).

The Hsf family in the model plant species, *Arabidopsis* and rice, consists of 21 and 25 members, respectively (Scharf *et al.*, 2012), divided into three classes: HsfA, HsfB, and HsfC. Among Hsf members, HsfA genes have been functionally characterized extensively in the model plant species, *Arabidopsis*, serving as *bona fide* transcription factors binding to HSEs. Despite the DNA-binding domain of the three classes of Hsf members being highly conserved, knowledge about the biological role of the HsfB and HsfC members is still scarce. It is generally recognized that HsfB and HsfC members do not contain an aromatic (W, Y, and F)–hydrophobic (L, I, V, and M)–acidic (D and E) residue-rich (AHA) activation domain present in HsfA members (Kotak *et al.*, 2004). HsfB members have been demonstrated to act as repressors (Ikeda and Ohme-Takagi, 2009; Ikeda *et al.*, 2011; Zhu *et al.*, 2012). Recently, Mittal *et al.* (2011) have shown that HsfB4b from rice is also capable of binding to HSE. The binding sequence of *Arabidopsis* HsfB1 has been shown to be different from that of HSEs; it binds to the TL1 element (GAAGAAGAA) and regulates pathogen defence genes (Pajerowska-Mukhtar *et al.*, 2012; Pick *et al.*, 2012). However, *Arabidopsis* HsfB1 can positively regulate the acquired thermotolerance (Ikeda *et al.*, 2011).

Some Hsf genes are constitutively expressed. A number of constitutively expressed HsfA1 proteins such as HsfA1a in tomato are known to be maintained in an inactive monomer state by association with Hsp90/Hsp70 under non-heat stress conditions (Hahn *et al.*, 2011). This complex is dissociated upon heat stress, which enables HsfA1a to form an active trimer. In *Arabidopsis*, constitutively expressed *HsfA1a* and *HsfA1b* are known to be the early response Hsf genes (Lohmann *et al.*, 2004; Busch *et al.*, 2005), serving as transcriptional activators for high-level induction of late response Hsf members such as *HsfA2*, which also plays an important role in acquired thermotolerance (Charng *et al.*, 2007).

In plants, there are some species-specific features in the role of individual Hsf members in regulating genes involved in the heat stress response. HsfA1a in tomato has been described as a master regulator for triggering the heat response and acquired thermotolerance (Mishra *et al.*, 2002). However, no master regulator has been found in *Arabidopsis* (Scharf *et al.*, 2012). Instead, three subclass A1 Hsfs (A1a, A1b, and A1d) in *Arabidopsis* are functionally redundant for triggering the heat response (Liu *et al.*, 2011; Liu and Charng, 2012). In tomato, HsfB1 functions as a transcription co-activator, cooperating with class A Hsfs during heat stress (Bharti *et al.*, 2004), while *Arabidopsis* HsfB1 was found to act as a repressor (Czarnecka-Verner *et al.*, 2004; Ikeda and Ohme-Takagi, 2009; Ikeda *et al.*, 2011; Zhu *et al.*, 2012). In addition, the compositions of Hsf classes differ markedly among plant species (Scharf *et al.*, 2012). Most notably, subclass C2 Hsfs appear to be monocot specific. However, the role of this subclass of Hsf in monocots is currently unknown. Conversely, subclasses A9, B3, and B5 are not present in monocot species (Scharf *et al.*, 2012). HsfA9 in sunflower and *Arabidopsis* is expressed specifically in seeds and controls Hsp expression during seed development (Almoguera *et al.*, 2002; Kotak *et al.*, 2007). In rice, HsfA7 appears to be expressed specifically in the seeds under normal conditions (Chauhan *et al.*, 2011*a*).

Investigation into the molecular basis of heat tolerance in wheat was pursued at the Hsp level as early as in the 1980s (Zivy, 1987; Krishnan *et al.*, 1989). Acquired thermotolerance in some thermotolerant wheat genotypes appears to be linked to higher transcript or protein levels of some Hsps (Vierling and Nguyen, 1992; Joshi *et al.*, 1997; Skylas *et al.*, 2002). More recently, Chauhan *et al.* (2012) have shown that overexpression of a small wheat chloroplastic Hsp (*TaHSP26*) in *Arabidopsis* improves thermotolerance. Comparative expression analysis between two genotypes with contrasting thermotolerance using the Affymetrix wheat genome array has shown that a large number of probe sets are differentially expressed in the heat-stressed leaves between the thermotolerant and thermosusceptible genotypes (Qin *et al.*, 2008). However, to date, no study has reported on the role of Hsf genes in regulating Hsp expression in wheat. Little is known about the Hsf family structure in wheat and the role of individual members in heat stress adaptation. Bread wheat (*Triticum aestivum*) is a hexaploid species and the Hsf family structure in wheat is expected to be more complex than that in diploid species. Only two members, HsfA4a and Hsf3 (HsfB2a), of the wheat Hsf family have been functionally analysed. *TaHsfA4a* is a cadmium-up-regulated gene and is involved in cadmium tolerance in wheat (Shim *et al.*, 2009). Overexpression of *TaHsf3* in transgenic *Arabidopsis* leads to enhanced thermotolerance and freezing tolerance (Zhang *et al*., 2013).

In this study, a genome-wide identification of the expressed members of the wheat Hsf family was performed using a bioinformatics analysis of currently available wheat sequences, including those isolated from the authors’ laboratory. A total of 56 members of the Hsf family from *T. aestivum* were identified. The wheat Hsf family structure was determined by phylogenetic analysis in accordance with the *Arabidopsis* and rice Hsf families. In order to understand their potential roles, the expression profiles of these Hsf members were investigated in wheat in various organs, in response to heat stress and other abiotic stresses (drought and salt stress). Functional analyses of representative wheat Hsf members were also performed to determine the functional HSE sequences in the promoter regions of four Hsp genes, to investigate whether they are capable of transactivating the expression of Hsp genes, and to identify with which elements they interact. Of particular note, it was demonstrated that a HsfC member was a transcriptional activator of Hsp genes; this has not been shown in any other plant species. These analyses provide some fundamental insights into the role of Hsfs in mediating wheat adaptation to heat stress through regulation of Hsp genes as well as their potential roles in adaptation to other major abiotic stresses.

## Materials and methods

### Plant materials and growth conditions

Spring wheat (*T. aestivum* L. cv. Bobwhite) plants were grown in a controlled-environment growth room in 1.5 litre pots, containing a 3:1:1 mix of sand:soil:peat under night/day conditions of 16h light (500 μmol m^–2^ s^–1^), 16/20 °C, and 80/60% relative humidity. For heat treatment experiments, Diatomite [silica gel-based artificial soil (0.5–2mm), Burnell Agencies, Brisbane, Australia] was used for plant growth for facilitating isolation of clean roots. Heat treatment of 1-month-old plants at 36 °C commenced at 2h after lights on, and the leaves and roots of heat-treated plants were harvested at 1.5h, 5h, and 3 d, with 5h of heat treatment per day. Control plants at 20 °C were harvested at the same time as for 1.5h heat-treated samples. All harvested samples were immediately immersed in liquid nitrogen and stored at –80 °C prior to RNA extraction.

### Identification and classification of Hsf members from *T. aestivum*

The sequences of Hsf DNA-binding domains from rice and *Arabidopsis* Hsf proteins in the Plant Transcription Factor Database (Riaño-Pachón *et al.*, 2007) were used for searching *T. aestivum* Hsf expressed sequence tags (ESTs) from the NBCI EST database and the assembled tentative consensus (TC) sequences from the *Triticum aestivum* Gene Index (TaGI) database (http://compbio.dfci.harvard.edu/tgi/). To increase the coverage of partial wheat Hsf cDNAs that lack sequences in the Hsf DNA-binding domain, full-length barley Hsf protein sequences retrieved from the NCBI sequence database were used to find additional wheat Hsf ESTs in the NCBI EST database. Unique Hsf representatives in wheat were identified by sequence alignment and contig assembly. After elimination of unreliable sequences at the ends of some poor quality ESTs, sequences that had >99.5% nucleotide identities were used for assembly into contigs. This analysis corrected many wrongly assembled Hsf TC sequences in the TaGI database. The assembled sequences were checked for correctness in the wheat genomic sequence database (Wilkinson *et al.*, 2012), and partial cDNAs were further extended with wheat genomic sequences. Many EST singletons which had no match with the wheat genomic sequence in the CerealsDB were discarded, as these singletons most probably represent poor sequence quality ESTs. To improve the coverage of the full-length Hsf cDNAs and the accuracy of Hsf cDNA assembly, the coding region sequences of 15 Hsf genes were also isolated using reverse transcription–PCR (RT–PCR). These cDNA sequences were then incorporated into the Hsf gene assembly. At the final stage, the wheat Hsf protein sequences from the assembled Hsf nucleotide sequences were checked by BLASTP research in the NCBI protein database for the presence of the Hsf DNA-binding domain and the partial Hsf sequences were checked for close homologues of barley full-length Hsf proteins.

Phylogenetic analysis was used to classify TaHsf members into three classes and further subclasses, based on the rice and *Arabidopsis* Hsf protein classification used by Scharf *et al.* (2012). Hsf DNA-binding domain and heptad repeat region (HR-A/B) sequences of Hsf proteins were used for generation of a phylogenetic tree by ClustalW alignment and the unrooted Neighbor–Joining method using MEGA 5.10 (Tamura *et al.*, 2011). Neighbor–Joining analysis was performed with pairwise deletion and Poisson correction. Bootstrap analysis was performed with 1000 replicates to assess the level of statistical support for each tree node. Partial TaHsf proteins that lack the DNA-binding domain and HR-A/B region were classified based on their highest sequence homology to full-length TaHsf members, but were excluded from the phylogenetic analysis.

### Total RNA extraction

Frozen samples were ground to fine powder in liquid nitrogen. Total RNA was isolated from samples using Plant RNA Reagent (Invitrogen, Carlsbad, CA, USA), according to the manufacturer’s instructions. RNA was further purified through a Qiagen RNeasy column (Qiagen, Australia) after pre-treatment with RNase-free DNase I (Xue and Loveridge, 2004).

### Isolation of *TaHsf* cDNAs

Wheat cDNAs were synthesized from total RNA prepared from the leaves and roots of wheat with or without heat treatment. Wheat Hsf cDNAs were isolated using RT–PCR or 3′-RACE (rapid amplification of cDNA ends) followed by PCR using primers designed from assembled Hsf sequences. The PCR-amplified products were cloned into pGEM-T Easy vector (Promega) and sequenced using a BigDye terminator cycle sequencing kit (Applied Biosystems, Foster City, CA, USA).

### Expression analysis using quantitative real-time PCR

The transcript levels of wheat genes were quantified from cDNA samples synthesized from DNase I-treated total RNA using real-time PCR with a ViiA™ 7 system (Applied Biosystems) and SYBR Green PCR Master Mix (Applied Biosystems) according to the manufacturer’s instructions. The sequences of primer pairs used for real-time PCR are listed in Supplementary Table S1 available at *JXB* online. The gene-specific primers were designed at the C-terminal domain or 3′-untranslated region. The gene specificity of primers for each gene during primer design was checked by blasting primer sequences in the TaGI database (http://compbio.dfci.harvard.edu/cgi-bin/tgi/gimain.pl?gudb=wheat) using an expect value setting at 10 000 and was also checked with all TaHsf DNA sequences assembled from this study to avoid matching to non-targeted genes.

Two wheat genes (*TaRPII36*, RNA polymerase II 36kDa subunit; and *TaRP15*, RNA polymerase I, II, and III, 15kDa subunit) were selected as internal reference genes for calculation of relative transcript levels of the genes under study (Xue *et al*., 2006, 2008*a*). The mRNA levels of these internal reference genes were similar in the control and heat-treated samples, as checked by the use of an external reference mRNA (626 nucleotides) *in vitro* transcribed from a bovine cDNA (CF767388), which was added to each RNA sample before cDNA synthesis (Xue *et al.*, 2011). The PCR efficiency of each primer pair was determined by a dilution series of samples. The specificity of real-time PCR amplification was confirmed by a single peak in melting temperature curve analysis of real-time PCR-amplified products. The apparent expression level of each gene relative to an internal reference gene (*TaRP15*) was calculated using the following formula: Er^Ct^/Et^Ct^×F (Stephenson *et al.*, 2007), where Ct is the cycle threshold (PCR cycle number of reference or target gene required for reaching the signal point used for detection across samples), Er is the reference gene (*TaRP15*) amplification efficiency, Et is the target gene amplification efficiency, and F is an amplicon size factor (reference gene amplicon size/target gene amplicon size). Apparent expression level values were tentatively used here to provide an approximate estimation of relative expression levels among various genes under the situation where the absolute quantification of mRNA levels for a large number of genes using a cRNA (or cDNA) calibration curve is not possible.

### Plasmid construction

pTaHsfA2b-CELD was constructed by translational fusion of the coding region sequence of *TaHsfA2b* to the N-terminus of the 6×His-tagged CELD reporter under the control of the *tac* promoter (Xue, 2005). *CelD* encodes a 1,4-β-glucanase (cellulase) from *Neocallimastix patriciarum* (Xue *et al.*, 1992). pUbiTaHsfA2b, pUbiTaHsfA4e, pUbiTaHsfB1b, pUbiTaHsfC1b, and pUbiTaHsfC2a plasmids were constructed by replacing *xylanase* in pUbiSXR (Vickers *et al.*, 2003) with the coding region of TaHsf cDNAs. TaHsfC2a mutant constructs (pUbiTaHsfC2a-mC1 and pUbiTaHsfCa2-mC2) were constructed using PCR and oligonucleotide primers containing the desired sequence mutation. *TaHsp17* and *TaHsp90.1-A1* promoter-driven *gfp* (green fluorescent protein) reporters were constructed by replacing the barley *HVA1* promoter in the *HVA1* promoter-driven *gfp* reporter gene (Xiao and Xue, 2001) with the PCR-amplified fragment of *TaHsp17* or *TaHsp90.1-A1* promoters, isolated in this study (see the Hsp promoter isolation section below). *TaHsp90.1-A*1 promoter mutants (psHsp90gfp and pΔHsp90gfp) were constructed using PCR-directed promoter truncation. The pHSE90gfp and pHSE17gfp constructs were made by adding three repeats of TaHsp90.1E1 or TaHsp17E1 elements to the *TaHsp90.1-A1* minimal promoter construct (pΔHsp90gfp).

### DNA-binding activity assays

For DNA-binding assays 6×His-tagged TaHsfA2b–CELD fusion protein was purified using Ni-NTA magnetic agarose beads (Qiagen, Australia) as described previously (Xue, 2005). Biotin-labelled double-stranded oligonucleotide probes were synthesized by filling in partially double-stranded oligonucleotides using the *Taq* polymerase reaction as follows: a 50 μl PCR containing 1× *Taq* polymerase buffer, 200 μM dNTPs, 150 pmol single-stranded oligonucleotide [5′-(sequence containing HSE or its mutants)–GAGTGGTGATTCCGGGCCTT (20bp linker sequence)], 150 pmol antisense biotin-labelled primer (5′-biotin-AAGGCCCG GAATCACCACTC) that was reverse complementary to the linker sequence, and 1U of *Taq* polymerase (Roche) with PCR cycling parameters as described previously (Xue *et al.*, 2006).

The DNA-binding activity of TaHsfA2b–CELD was measured as described previously (Xue, 2002*a*) using StreptaWell High Bind (streptavidin-coated 96-well plates from Roche) and binding/washing buffer [25mM HEPES/KOH, pH 7.0, 50mM KCl, 2mM MgCl_2_, and 0.5mM dithiothreitol (DTT) containing 0.15 μg μl^–1^ shared herring sperm DNA, 0.3mg ml^–1^ bovine serum albumin, 10% glycerol, and 0.025% NP-40. About 50000 fluorescent units h^–1^ of the CELD activity of TaHsfA2b–CELD fusion protein and 0.4 pmol of biotinylated probes were used per assay. The cellulase activity of the CELD fusion proteins bound to immobilized biotinylated probes was assayed by incubation in 100 μl of the CELD substrate solution (1mM methylumbelliferyl-β-d-cellobioside in 50mM Na-citrate buffer, pH 6.0) at 40 °C for 4h. DNA-binding assays with a biotin-labelled double-stranded oligonucleotide without a HSE were used as a control of background activity.

### Transactivation assays

Transactivation assays were performed as described previously (Xue, 2003). Constructs were introduced into the seedlings of wheat (cv. Bobwhite) by particle bombardment (Xue *et al.*, 2003). The effector gene was co-introduced with a *gfp* reporter gene driven by *TaHsp17,TaHsp90.1-A1*, or its derivative promoter to determine the transactivation activity. The *gfp* reporter gene without a TaHsf effector gene was used as a control. A β-glucuronidase (GUS+) reporter driven by the maize *Ubi1* promoter, as described by Vickers *et al.* (2003), was also co-bombarded for validation of transformation events among assays. The bombarded seedlings were kept at room temperature (22 °C) or 36 °C in the dark for ~20h. GFP foci were examined under a fluorescence microscope (Xue, 2002*b*). The tissue sections that had GFP foci were subsequently stained for histochemical detection of GUS activity according to the method of Jefferson (1987).

### Isolation of TaHSP promoter sequences

The promoter sequences of *TaHsp17* and *TaHsp90.1-A1* were isolated with PCR amplification of genomic DNA of *T. aestivum* genotype SB169 (Xue *et al.*, 2008*b*). PCR primers were designed based on the assembled sequences through extension of the *TaHsp17* and *TaHsp90.1-A1* EST or cDNA sequences using the wheat genome sequence database in CerealDB (Wilkinson *et al.*, 2012). The PCR-amplified DNA fragments were cloned and sequenced. Two promoter sequences were deposited in GenBank [*TaHsp17* promoter (1214bp upstream of the translation start codon), KF208539; and *TaHsp90.1-A1* promoter (1375bp upstream of the translation start codon), KF208540].

### Expression analysis of Affymetrix GeneChip wheat genome array data sets

The wheat genome array (Affymetrix GeneChip) contains 61 127 probe sets representing 55052 transcripts for all 42 chromosomes in the wheat genome. The raw GeneChip data of TA23 and E-MEXP-971 were retrieved from http://www.plexdb.org and http://www.ebi.ac.uk/arrayexpress/ as cel files, respectively. The Affymetrix data sets were analysed as described previously (Xue *et al.*, 2013), using GeneChip robust multiarray average (GC-RMA) for normalization (Wu *et al.*, 2004). Normalized values were converted to non-log values for comparative analysis of TaHsf genes between treatments. Probe sets with normalized hybridization signals of <20 were considered as not detectable.

## Results

### Fifty-six members were identified from the TaHsf family

Fifty-six TaHsf members were identified through a combination of sequence analysis of wheat EST and nucleotide collection databases in NCBI (http://blast.ncbi.nlm.nih.gov/), followed by confirmation and sequence extension analyses using wheat genome sequences in the CerealDB (http://www.cerealsdb.uk.net/CerealsDB/Documents/DOC_search_reads.php, Wilkinson *et al.*, 2012), and isolation of 15 TaHsf cDNAs in this study (Table 1). Sequence IDs used for assembly of these TaHsf members are listed in Supplementary Table S2 at *JXB* online. The deduced protein sequences of 56 TaHsf genes are shown in Supplementary Fig. S1. Of these 56 genes, 39 TaHsf genes contain a full-length coding region sequence. A number of these partial TaHsf proteins identified contain half a Hsf DNA-binding domain. There is a very large intron between the LNTY and GFRK sequence in the DNA-binding domain, and this makes successful extension of partial ESTs difficult using the incomplete wheat genome sequence database in the CerealDB. It is possible that two partial cDNAs, TaHsfA2i and TaHsfA6f, containing an LNTY half-sequence may be a part of GFRK-half cDNAs (Supplementary Fig. S2). The multiple sequence alignments of the DNA-binding domains and heptad repeats (HR-A core and HR-B) of TaHsf proteins are shown in Supplementary Figs S2 and S3, respectively, illustrating highly conserved amino residues in the DNA-binding domain and primary sequence features in the HR-A core/B region in these TaHsf proteins.

**Table 1.** The list of TaHsf members identified

Gene	Full or partial (C or N) ORF	No. of amino acids	pI	Mol. wt (kDa)	Affymetrix probe set ID (no. of probe matches)^*a*^
TaHsfA1a	Full	529	4.99	58.1	
TaHsfA1b	Full	522	4.91	57.5	Ta.8059.1.S1_at (7)
TaHsfA1c	Almost full	NA	NA	NA	
TaHsfA2a	Full	353	5.36	39.7	TaAffx.92707.2.S1_at (11)
TaHsfA2b	Full	413	4.99	45.6	Ta.6737.2.S1_a_at (11), TaAffx.105519.1.S1_at (8)
TaHsfA2c	Full	404	5.06	44.8	TaAffx.105519.1.S1_at (8)
TaHsfA2d	Partial (C)	NA	NA	NA	TaAffx.92707.1.S1_at (9)
TaHsfA2e	Partial (C)	NA	NA	NA	Ta.6737.2.S1_a_at (11)
TaHsfA2f	Partial (C)	NA	NA	NA	Ta.1276.1.A1_at (10)
TaHsfA2g	Partial (N)	NA	NA	NA	
TaHsfA2h	Full	405	5.06	44.9	TaAffx.105519.1.S1_at (8)
TaHsfA2i	Partial (N)	NA	NA	NA	
TaHsfA3a	Full	499	5.7	54.8	TaAffx.12296.1.S1_at (11)
TaHsfA3b	Partial (C)	NA	NA	NA	
TaHsfA4a	Full	432	5.36	48.4	
TaHsfA4b	Full	441	5.18	49.5	
TaHsfA4c	Full	448	4.91	50.2	TaAffx.120360.1.A1_at (4)
TaHsfA4d	Full	442	5.11	49.7	TaAffx.5995.1.S1_at (8), TaAffx.120360.1.A1_at (5)
TaHsfA4e	Full	445	4.93	49.9	TaAffx.120360.1.A1_at (11)
TaHsfA4f	Partial (C)	NA	NA	NA	
TaHsfA5a	Full	458	5.21	49.9	
TaHsfA5b	Full	455	5.33	49.9	
TaHsfA6a	Full	341	5.02	39.5	Ta.27873.1.A1_at (9)
TaHsfA6b	Partial (C)	NA	NA	NA	
TaHsfA6c	Partial (C)	NA	NA	NA	Ta.28772.1.S1_at (6)
TaHsfA6d	Partial (C)	NA	NA	NA	Ta.28772.1.S1_at (9)
TaHsfA6e	Full	370	5.03	42	Ta.28772.1.S1_at (6)
TaHsfA6f	Partial (N)	NA	NA	NA	
TaHsfA7a	Full	353	4.79	38.2	
TaHsfA7b	Full	351	4.83	38.2	
TaHsfA8a	Full	384	5.15	43.1	TaAffx.40092.1.S1_at (7)
TaHsfA8b	Partial (C)	NA	NA	NA	TaAffx.40092.1.S1_at (5)
TaHsfA8c	Partial (C)	NA	NA	NA	TaAffx.40092.1.S1_at (11)
TaHsfB1a	Full	298	9.5	32.2	Ta.11671.1.S1_at (9)
TaHsfB1b	Full	298	9.31	32.3	
TaHsfB1c	Full	298	9.2	32.1	Ta.11671.2.S1_x_at (6)
TaHsfB2a	Full	314	9.46	34.7	
TaHsfB2b	Full	374	5.44	40.4	
TaHsfB2c	Full	397	4.89	41.4	
TaHsfB2d	Full	396	4.89	41.1	
TaHsfB2e	Full	397	5.28	41.3	Ta.18067.2.S1_x_at (4)
TaHsfB4a	Full	320	6.55	35.3	
TaHsfB4b	Partial (N)	NA	NA	NA	
TaHsfB4c	Partial (N)	NA	NA	NA	
TaHsfC1a	Full	325	6.08	35.8	
TaHsfC1b	Full	236	6.91	26.1	Ta.8266.1.A1_s_at (11), TaAffx.34778.1.S1_at (11)
TaHsfC1c	Full	234	7.65	25.8	Ta.8266.1.A1_s_at (7)
TaHsfC1d	Full	241	8.76	26.4	Ta.8266.1.A1_s_at (11)
TaHsfC1e	Full	325	5.94	35.7	
TaHsfC2a	Full	263	6.11	28.0	Ta.5852.1.A1_at (9), Ta.5852.1.A1_x_at (9)
TaHsfC2b	Full	270	6.92	28.7	
TaHsfC2c	Almost full	NA	NA	NA	
TaHsfC2d	Full	274	6.98	30	Ta.13964.1.S1_at (11)
TaHsfC2e	Full	276	6.46	30.3	
TaHsfC2f	Full	264	5.43	29.1	Ta.13964.1.S1_at (11)
TaHsfC2g	Full	275	8.4	30.2	Ta.25627.1.A1_at (11), Ta.25627.1.A1_x_at (11)

N, the N-terminal part; C, the C-terminal part; NA, not applicable.

^*a*^ The Affymetrix wheat genome array has 11 probes for each probe set ID.

### Phylogenetic analysis and classification of TaHsf proteins

**Fig. 1.**
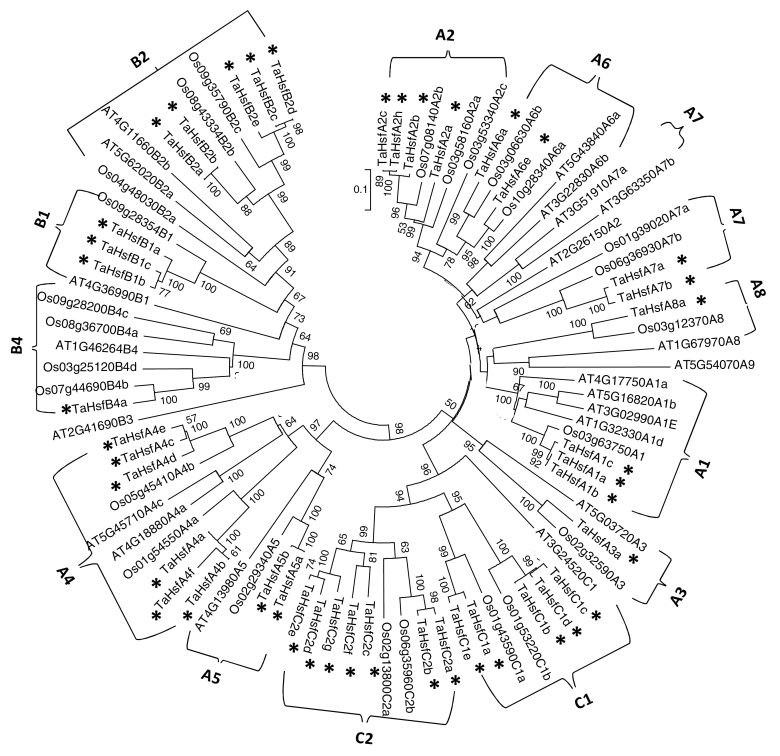
The Neighbor–Joining phylogenetic tree of Hsf proteins from wheat, rice, and *Arabidopsis*. The N-proximal regions (from the start of the DNA-binding domain to the end of the HR-A/B region) of Hsf proteins were used for construction of the phylogenetic tree using the MEGA 5.10 program. Unrooted Neighbor–Joining analysis was performed with pairwise deletion and Poisson correction. For rice (prefixed by Os) and *Arabidopsis* (prefixed by AT) Hsf proteins, both locus ID and subclass number were used (e.g. AT4G17750A1a=AtHsfA1a with a locus ID AT4G17750). TaHsf proteins are marked with asterisks. Bootstrap values >50 are shown.

In order to gain some insight into the potential function of TaHsf proteins from known function Hsfs in model plant species (*Arabidopsis* and rice), phylogenetic analysis of Hsf proteins from wheat, rice, and *Arabidopsis* was performed using the sequence region from the start of DNA-binding domain to the end of the HR-A/B region (Fig. 1). Class and subclass classification of TaHsf proteins is based on those of *Arabidopsis* and rice Hsf proteins as reported by Scharf *et al.* (2012). The phylogenetic tree of the TaHsf family members is shown in Supplementary Fig. S4 at *JXB* online. From Fig. 1, it can be seen that while class B and C Hsf members form distinct groups, class A Hsf protein members form at least three smaller clusters; two of the class A clusters appear to be more similar to class C Hsf proteins than to the third class A cluster. Class C Hsf proteins appear to be more similar to class A Hsfs, compared with class B members. Wheat Hsf proteins cluster more closely with rice Hsfs than with *Arabidopsis* Hsfs. *Arabidopsis* A2 is not clustered with the monocot A2 group. Wheat Hsf A, B, and C classes contain eight, three, and two subclasses, respectively, which are the same as those in the rice Hsf family. Subclasses A9 and B3 are absent in wheat and rice, and subclass C2 is unique in monocot species. TaHsf members with an incomplete sequence of the DNA-binding domain and HR-A/B region were subsequently classified based on their closest sequence homology with TaHsf members containing a complete sequence. In comparison with the *Arabidopsis* Hsf family, the wheat Hsf family contains a large number of A2 group members (Table 1), in addition to the differences in the A9, B3, and C2 groups.

### Organ distribution of TaHsf transcripts

**Fig. 2.**
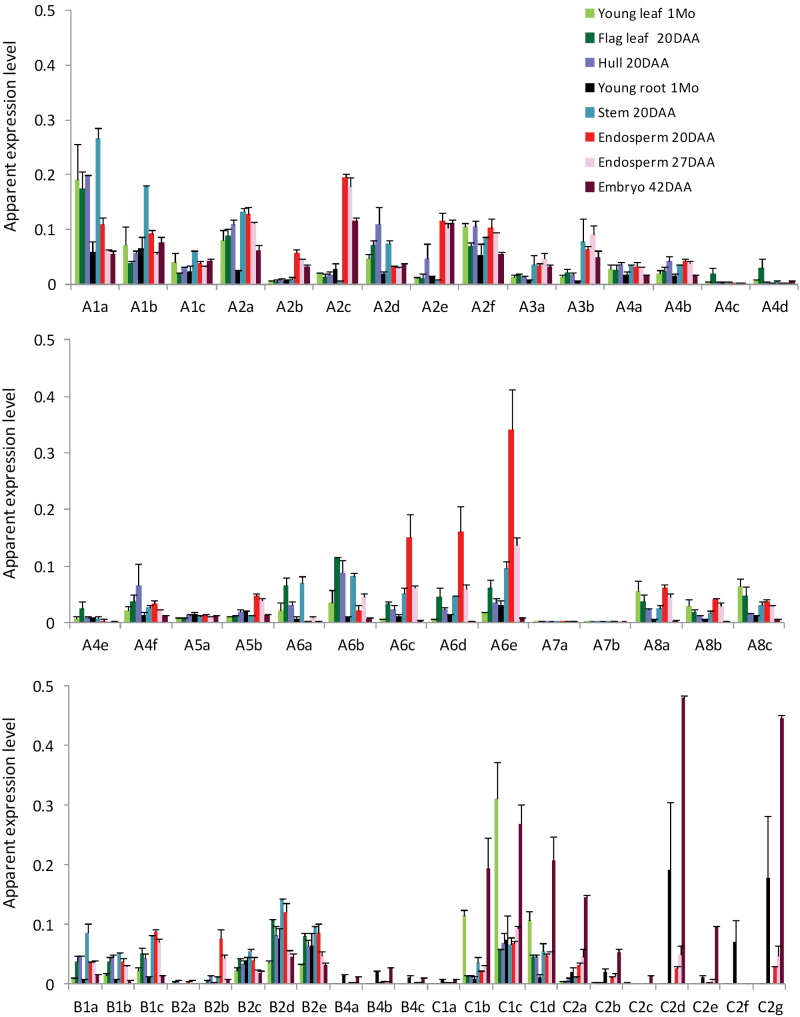
Relative mRNA abundance of TaHsf members in wheat organs. Values are means ±SD of three biological replicates and are expressed as apparent expression levels relative to a control gene *TaRP15*. 1Mo, 1-month-old; 20DAA, 20 d after anthesis; 27DAA, 27 d after anthesis; 42DAA, 42 d after anthesis.

In order to understand the biological roles of individual TaHsf members in wheat, their relative transcript distribution was surveyed under non-stress conditions in six major organs (leaf, root, stem, hull, endosperm, and embryo), with leaf and endosperm studied at two developmental stages. Real-time PCR primers targeted to the 3′ regions or C-terminal domain region of cDNAs for TaHsf members were designed with full-length cDNA or containing full or partial C-terminal part sequences, with the exception of *TaHsfA2h*, which was isolated after completion of expression analysis. The mRNA levels of individual members are presented as apparent expression levels relative to the internal control gene, *TaRP15* (Fig. 2), which are used for the assessment of relative expression levels between genes (Stephenson *et al.*, 2007). TaHsf subclass A1 group genes were constitutively expressed at relatively high levels among the six organs. Other class A genes constitutively expressed at relatively high levels in the green organs examined (leaf, stem, and hull) were some of the A2, A6, and A8 members. Class A members that were predominantly expressed in the endosperm were A2b, A2c, A2e, A5b, A6c, A6d, and A6e, and the apparent expression levels of many of these endosperm-predominantly expressed members were higher than that of the A1 members in the endosperm. In particular, the constitutive expression of A6e in the endosperm was at least three times higher than that of the A1 group genes. A6e is highly homologous to a partial TaHsf cDNA (GD186916) isolated from the *T. aestivum* developing seed heat stress forward subtractive library, which has been shown to have a seed-predominant expression pattern (Chauhan *et al.*, 2011*b*). Two A2 members (A2c and A2e) were expressed in the maturing embryo at a higher level than the A1 members.

Some class B members (B2c, B2d, and B2e) were also constitutively expressed in these six organs at a relatively high level (Fig. 2). The subclass B1 members were expressed at higher levels in organs at the reproductive stage than in young leaves and roots. The expression of B4 members was mainly confined to the roots and embryos under normal conditions. The transcript distribution of class C members in these organs had the following features: (i) several C1 members (C1b, C1c, and C1d) were highly expressed in the young leaves and embryos, with the apparent expression level of C1c in the young leaves higher than those of the A1 group; and (ii) C2 members were predominantly expressed in embryos, and their apparent expression levels in embryos and roots were much higher than any other subclasses of TaHsf genes.

**Fig. 3.**
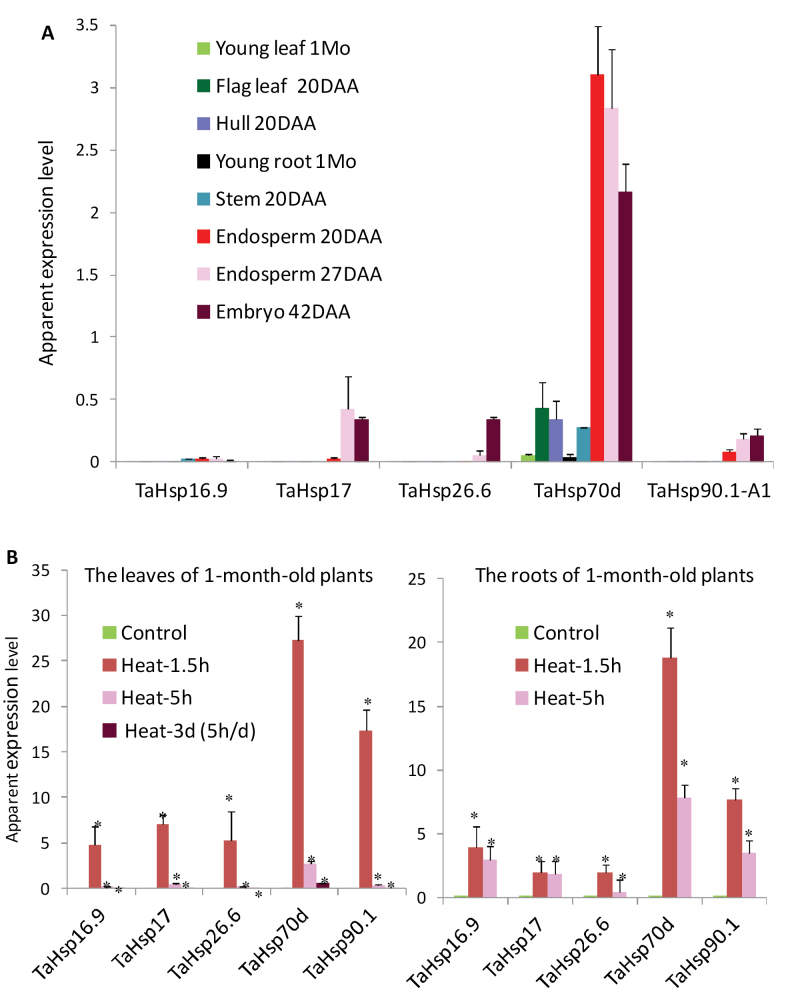
Expression profiles of TaHsp genes. Values are means ±SD of three biological replicates and are expressed as apparent expression levels relative to a control gene *TaRP15*. (A) Organ mRNA distribution. (B) Response to heat stress in the leaves and roots. Statistical significance in differences between control and heat-treated groups (36 °C for 1.5h, 5h, or 3 d with 5h d^–1^) is indicated by an asterisk.

To understand the functionality of TaHsf proteins under normal conditions, some representative Hsp genes were also analysed. Although many class A members were constitutively expressed in vegetative organs, many Hsp genes (*TaHsp16.9*, *TaHsp17*, *TaHsp26.6*, and *TaHsp90.1-A1*) were not expressed or were expressed at very low levels in these vegetative organs under normal conditions (Fig. 3A). However, many of these wheat Hsp genes, particularly *TaHsp70d*, were constitutively expressed at a high level in endosperms and maturing embryos.

### Subclasses A2, A6, and B2 members become predominant TaHsf transcripts during heat stress

**Fig. 4.**
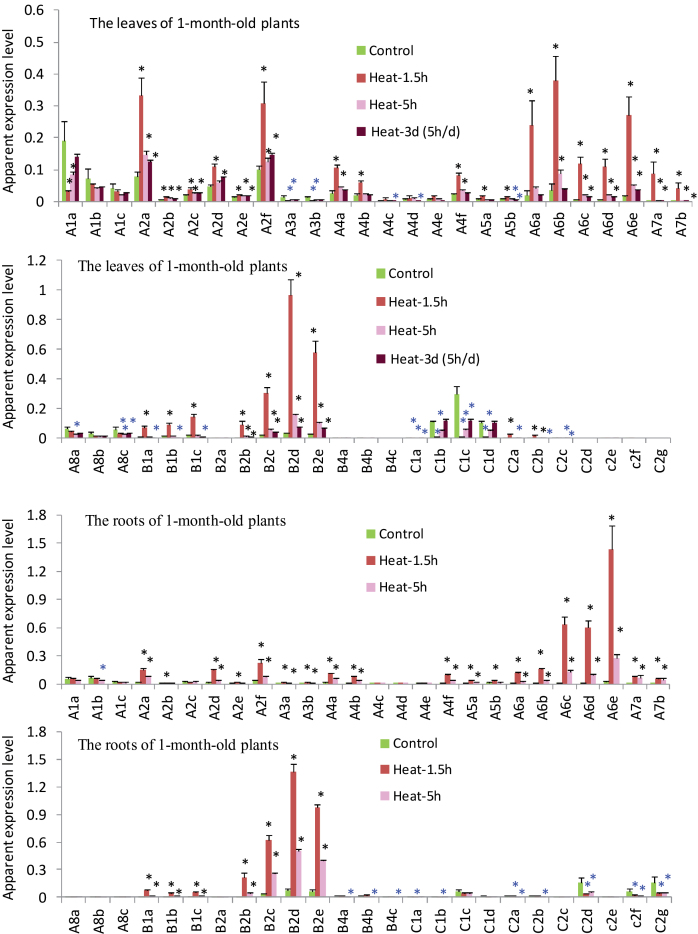
Expression changes of TaHsf members during heat stress. Values are means ±SD of three biological replicates and are expressed as apparent expression levels relative to a control gene *TaRP15*. Statistical significance in differences between control and heat-treated groups (36 °C for 1.5h, 5h, or 3 d with 5h d^–1^) is indicated by a black (up-regulated) or blue (down-regulated) asterisk. (Note: the expression levels of some TaHsfs are too low to be seen in this comparative presentation, but statistical significance in the changes of their transcript levels in response to heat stress is shown.)

To provide a heat response profile for individual TaHsf members, the heat responsiveness of these genes in the leaves and roots of 1-month-old plants with short- (1.5h and 5h) and long-term (3 d with 5h heat treatment each day) heat stress (at 36 °C) was examined. The expression of subclass A1 group members was not up-regulated during heat stress; in fact, the A1a mRNA level was down-regulated in leaves with a short-term heat treatment and a marked reduction in its mRNA level was observed in the 1.5h treatment (Fig. 4). Transcript down-regulation by heat in the leaves was also observed in the A3 group, A4c, A4d, A8a, and A8c genes. Heat up-regulation was observed in all A2, A6, and A7 members, as well as in some members of the A4 and A5 groups. In the early heat treatments, the A2 and A6 groups were the predominantly expressed HsfAs in the leaves. In the roots of heat-stressed plants, the A6 group genes were the predominantly expressed HsfA genes. In particular, the A6e mRNA level in the roots in the 1.5h heat treatment was ~25 times higher than that of any of the A1 members.

Subclass B1 and B2 members were also highly heat inducible with the 1.5h treatment (Fig. 4). However, in contrast to B2 members, heat induction of the B1 group in the leaves was transient, and repeated heat stress for 3 d resulted in down-regulation of their transcript levels. The transcript levels of the B2 group during heat stress were the highest among TaHsf subclasses. The B4 group was not heat inducible and their transcript levels in the roots of plants with repeated heat stress were significantly lower than in the non-stress control plants. The C1 group, which were expressed in the leaves under non-stress conditions at very high levels compared with other TaHsf groups, generally showed rapid down-regulation in the early heat treatment (1.5h), particularly in the leaves, and their transcript levels gradually returned back to the control levels with prolonged heat treatment. Most C2 members, which were not expressed in the leaves but were expressed at high levels in the control roots, were also down-regulated by heat stress. However, C2a and C2b expression was inducible in the leaves by >5-fold with a 1.5h heat treatment, but was down-regulated after repeated heat stress for 3 d.

Interestingly, heat induction of TaHsf genes was rapidly attenuated in the 5h treatment, and for many TaHsf genes further reduction was seen in samples repeatedly heat treated for 3 d (5h d^–1^). This heat response pattern was also observed in the TaHsp genes examined (Fig. 3B).

### A number of TaHsf subclasses are up-regulated during drought and salt stress

**Fig. 5.**
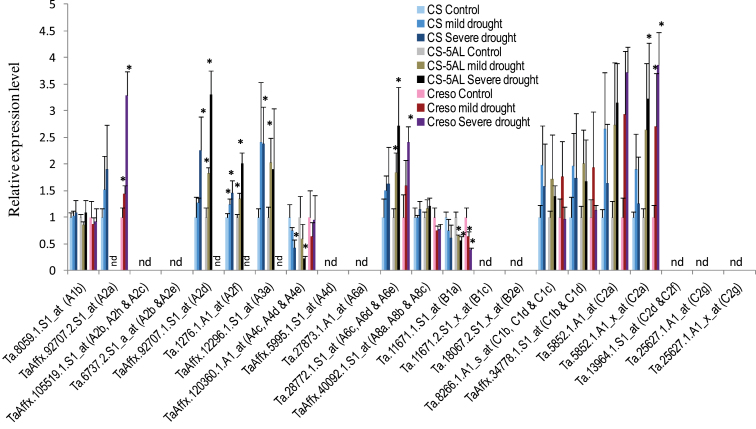
Drought responsiveness of TaHsf genes in the flag leaves and glumes of *T. aestivum* Chinese Spring (CS) and *T. durum* Creso. Raw data are derived from an Affymetrix wheat genome array data set at http://www.plexdb.org (accession # TA23; Aprile *et al.*, 2009). Values are means ±SD of three biological replicates and relative expression levels within each genotype (stress groups versus control group and each control group was arbitrarily set as 1). 5AL, Chinese Spring 5A deletion line. Hybridization signal <20 is considered not detectable (nd). *P < 0.05.

**Fig. 6.**
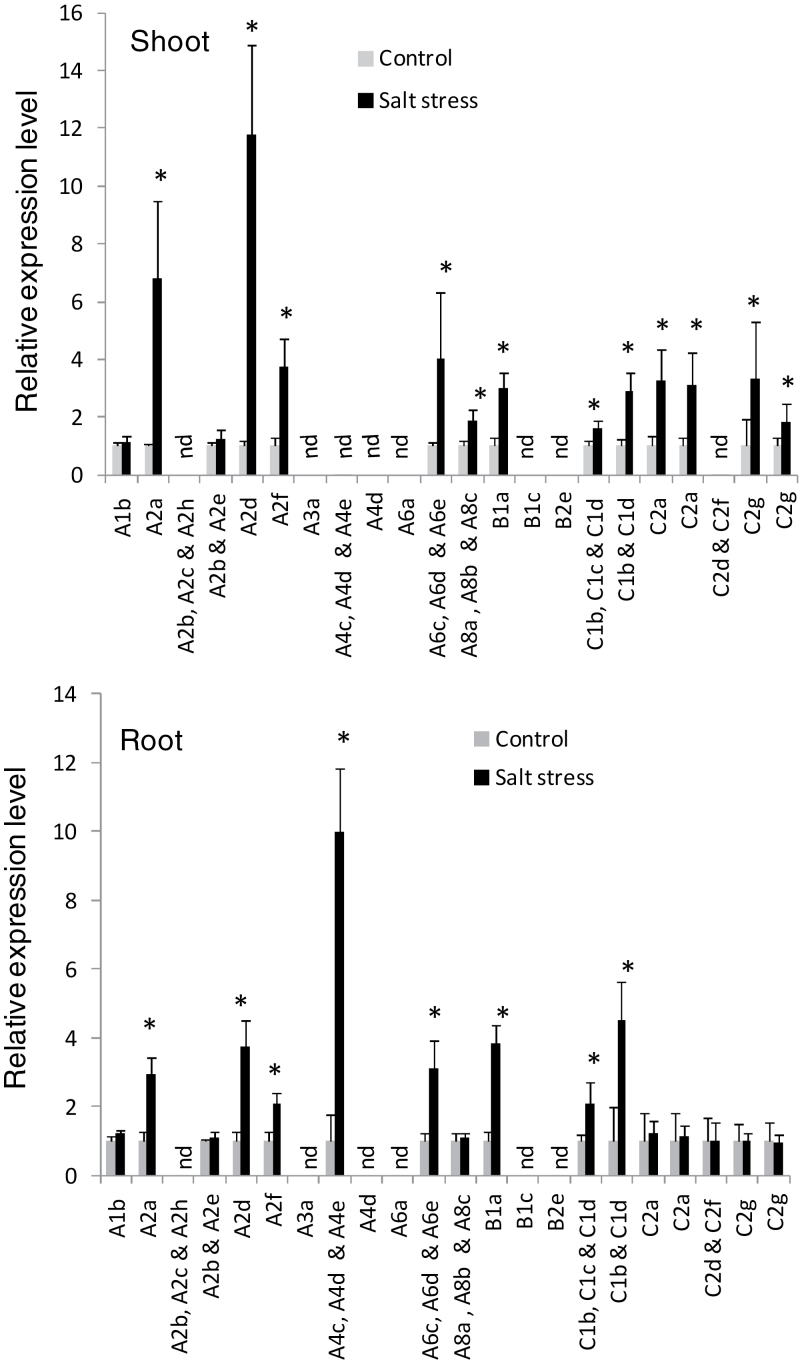
Expression changes of TaHsf genes in the shoot and roots of bread wheat plants in response to long-term salt stress. Raw data are derived from Affymetrix wheat genome array at http://www.ebi.ac.uk/arrayexpress/ (accession #: E-MEXP-971; Mott and Wang, 2007). Values are means ±SD of 15 biological replicates and are expressed as relative expression level (stress group versus control group and each control group was arbitrarily set as 1). The Affymetrix probe sets for these genes are in the same order as shown in Fig. 5. Hybridization signal <20 is considered not detectable (nd). *P < 0.01.

Although the major role of Hsfs is known to be the regulation of heat-responsive genes involved in heat acclimatization, it was also of interest to see whether this family is involved in wheat responses to other major abiotic stresses such as drought and salt stress. Using the nucleotide sequences of these identified TaHsf members to search probe sets in the Affymetrix wheat genome array at the NetAffx Analysis Center of the Affymetrix website (http://www.affymetrix.com/index.affx), 22 probe set IDs that have at least four matches out of 11 probes per probe set ID were identified (Table 1). However, eight probe set IDs cross-hybridize with more than one TaHsf gene, and two genes (C2a and C2g) are represented with each having two probe set IDs (Fig. 5). There are two publically deposited Affymetrix data sets, TA23 (http://www.plexdb.org; Aprile *et al.*, 2009) and E-MEXP-971 (http:/www.ebi.ac/arrayexpress/; Mott and Wang, 2007), for transcript profiling in wheat responses to drought and salt stresses. The TA23 data set investigates expression changes in the flag leaves and glumes (two organs pooled) of hexaploid wheat (*T. aestivum*) and tetraploid wheat (*T. turgidum* subsp. *durum*) at the grain-filling stage during mild or severe drought stress in comparison with control plants. Among these Hsf probe sets, 13, 12, and 10 probe sets had detectable hybridization signals in the flag leaf and glume tissues of *T. aestivum* cv. Chinese Spring, the Chinese Spring-5A deletion line, and durum wheat, respectively. Of these detectable probe sets, the A2, A3, A6, C1, and C2 groups generally showed increased expression during drought stress. In contrast, the transcript levels of the A4 and B1 groups were down-regulated by drought. The drought-mediated expression level changes (<4-fold) of these Hsf genes were not as great as those seen in the short-term heat stress (Fig. 4), but were comparable with those during long-term heat stress (3 d with 5h d^–1^). The transcript level changes of these TaHsf genes in the shoots and roots of *T. aestivum* plants after long-term salt stress from the E-MEXP-971 Affymetrix data set are shown in Fig. 6. In this data set, five genotypes were placed (each with three replicates) with the same treatment in the same group to increase the number of biological replicates for analysis. A total of 12 and eight probe sets with a significant increase in expression were observed in the salt-treated shoots and roots, respectively. The up-regulated TaHsf genes were A2, A6, A8, B1, C1, and C2 groups in the shoot and A2, A4, A6, and C1 groups in the root. Some of these increases in the expression levels in salt-stressed plants were remarkable. A >10-fold increase in the A2d mRNA level was seen in the salt-stressed shoot, and a 10-fold increase in the A4 group expression levels was observed in the salt-stressed root.

### TaHsfA2b, A4e, and C2a are transcriptional activators of heat shock protein genes

**Fig. 7.**
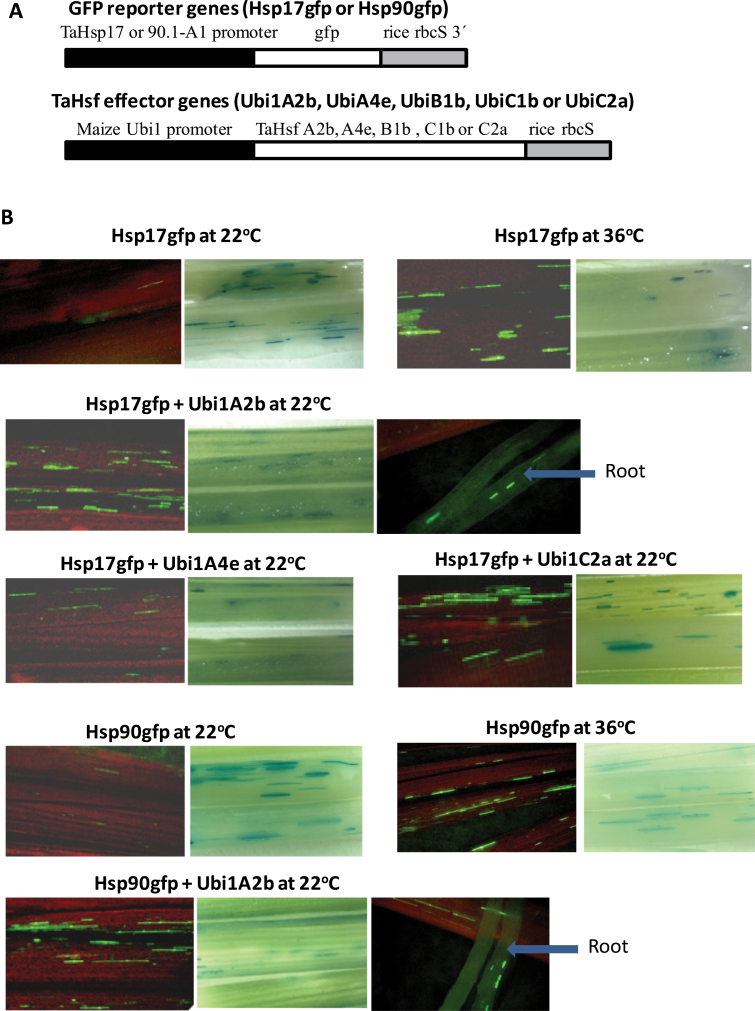
Transactivation of TaHsp promoter-driven reporter genes by TaHsfs. (A) Reporter and effector gene constructs. (B) Transactivation of the *TaHsp17* or *TaHsp90.1-A1* promoter-driven *gfp* reporter gene by a constitutively expressed *TaHsfA2b*, *A4e*, or *C2a* effector gene. The maize *Ubi1* promoter-driven GUS reporter was co-bombarded with test constructs to check transformation events. GFP foci (green) indicate the expression of the TaHsp promoter-driven *gfp* reporter gene, and blue foci resulted from the expression of the co-introduced GUS reporter as indication that tissue sections were transformed with these constructs. The red background is the red fluorescence of shoot chlorophyll. The *TaHsfA4e* or *TaHsfC2a* effector gene also activated the expression of the Hsp90gfp reporter gene (data not shown). Co-bombardment of a *TaHsfB1b* or *TaHsfC1b* effector gene with a Hsp17gfp or Hsp90gfp reporter gene did not produce GFP foci (data not shown).

To investigate the regulatory role of TaHsf transcription factors in controlling the expression of Hsp genes in wheat, transactivation assays were performed in wheat seedlings using the promoters of *TaHsp17* and *TaHsp90.1-A1* to drive the expression of *gfp* as reporter genes, and a constitutively expressed maize *Ubi1* promoter to drive the expression of five TaHsf members from A2, A4, B1, C1, and C2 subclasses (A2b, A4e, B1b, C1b, and C2a) as effector genes. *TaHsp17* and *TaHsp90.1-A1* promoter fragments were isolated based on the assembled sequences obtained through analysis of the wheat genome sequence database to extend the sequences of *TaHsp17* (CD909515 and CJ523462) and *TaHsp90.1-A1* (HX149366 and GQ240772) cDNA or ESTs. A control GUS reporter gene driven by the maize *Ubi1* promoter was used for checking the transformation events across the samples. Quantitative real-time PCR analysis showed that the mRNA levels of *TaHsp17* and *TaHsp90.1-A1* were extremely low in wheat leaves and roots under non-stress conditions (Fig. 3). Green foci of the *gfp* reporter genes driven by the *TaHsp17* or *TaHsp90.1-A1* promoter were rarely found in wheat seedling shoots and roots incubated at 22 °C, when they were bombarded into these seedlings without a TaHsf effector gene (Fig. 7). When the reporter gene-bombarded seedlings were incubated at 36 °C, many strong GFP foci were observed (Fig. 7), indicating that expression of the *TaHsp17* or *TaHsp90.1-A1* promoter-driven reporter gene was induced by heat treatment. To confirm that the 22 °C samples bombarded with the *TaHsp17* or *TaHsp90.1-A1* reporter gene were transformed, tissue sections where GFP foci could be found were selected for histochemical staining of GUS activity, and many strong GUS foci were observed in the samples incubated at 22 °C (Fig. 7), confirming that these *gfp* reporter genes were essentially not expressed in the shoots and roots of wheat seedlings at 22 °C. Introduction of these reporter genes with a TaHsf effector gene (A2b, A4e, or C2a) resulted in induction of GFP foci in the shoots and roots of seedlings at 22 °C (Fig. 7; the activation pictures of the *TaHsp90.1-A1* reporter gene by HsfA4e or HsfC2a effector gene are not illustrated), indicating that *TaHsp17* and *TaHsp90.1-A1* promoters were activated by a TaHsf transcriptional activator. GFP foci in the samples co-transformed with a *TaHsfA2b* effector gene were stronger than those with a *TaHsfA4e* effector gene. Interestingly, GFP foci in the samples co-transformed with a *TaHsfC2a* effector gene were as strong as those with the *TaHsfA2b* effector gene. There was no induction of the *TaHsp17*- or *TaHsp90.1-A1*-driven reporter gene expression when *TaHsfB1b* or *TaHsfC1b* was used as an effector gene (data not shown).

### TaHsfA2b binds to *cis*-acting elements in the promoters of heat shock proteins

**Fig. 8.**
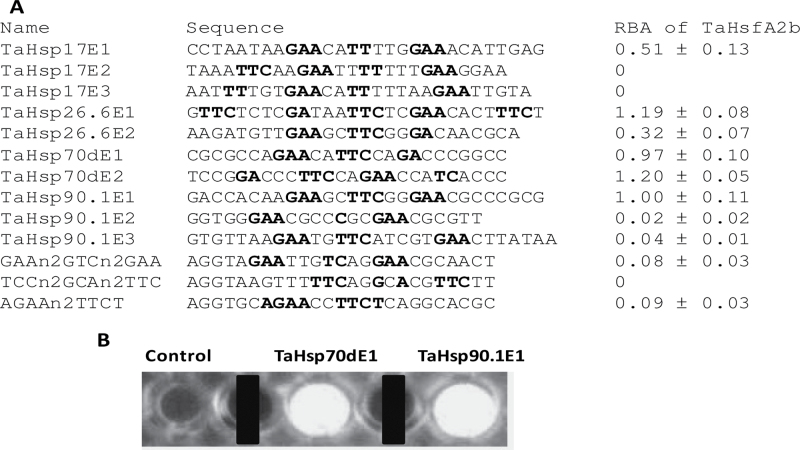
TaHsfA2b binding to elements present in the promoters of *TaHsp17*, *TaHsp26.6*, *TaHp70d*, and *TaHsp90.1-A1* genes. (A) Relative binding activity (RBA) of TaHsfA2b to nGAAn or nTTCn sequences. Values are means ±SD of three assays and are relative to the binding activity of TaHsp90.1E1, which is arbitrarily set as 1. GAA or TTC sequences are in bold. TaHsp sequences are selected from the available promoter region sequences within 1.5kb upstream of the translation start of these TaHsp genes. Three artificial sequences (GAAn2GTCn2GAA, TCCn2GCAn2TCC, and AGAAn2TTCT) were also tested for tolerance of nucleotide sequences deviating from the perfect HSE (nGAAnnTCCnnGAA or nTCCnnGAAnnTCCn). (B) Illustration of TaHsfA2b binding activity as the 4-methylumbelliferyl group fluorescence produced through hydrolysis of methylumbelliferyl-β-d-cellobioside by TaHsfA2b–CELD that bound to immobilized HSE-containing oligonucleotides (TaHsp70dE1 and TaHsp90.1E1). Control is the oligonucleotide containing no HSE elements.

Transactivation of *TaHsp17* and *TaHsp90.1-A1* genes by the TaHsfA members suggests that these Hsp genes are their target genes. To investigate further whether this transactivation is direct or indirect, whether the class A group was capable of directly binding to the *cis*-acting elements in the promoters of heat-inducible Hsp genes was studied using the TaHsfA2b protein. The available promoter sequences within 1.5kb upstream of the translation start of four Hsp genes (*TaHsp17*, *TaHsp90.1-A1*, *TaHsp26.6*, and *TaHsp70d*) were analysed for the presence of HSEs. *TaHsp26.6* and *TaHsp70d* promoter region sequences were also identified through analysis of the wheat genome sequence database to extend the sequences of *TaHsp26.6* (AF097659) and *TaHsp70d* (AF005993) cDNAs (assembled *TaHsp26.6* and *TaHsp70d* promoter region sequences are shown in Supplementary Fig. S5 at *JXB* online). A typical HSE (5′-nGAAnnTTCnnGAAn or 5′-nTCCnnGAAnnTTCn) was found only in the *TaHsp90.1-A1* promoter and was named TaHsp90.1E1. Therefore, some sequences containing at least two nGAAn inverted repeats or three nGAAn inverted repeats with gaps or one nucleotide degeneracy in one repeat were retrieved for testing of their binding by TaHsfA2b protein *in vitro* using a CELD fusion system (Xue, 2002*a*). As shown in Fig. 8, TaHsfA2b binds to many of the sequences selected from these four promoters with varying affinity. At least one functional HSE was found in each of these four promoters. Some atypical HSE sequences (TaHsp26.6E1 and TaHsp70dE2) had even higher TaHsfA2b binding affinity than TaHsp90.1E1. TaHsp90.1E3 with an additional three nucleotides in one spacer of three nGAAn/nTTCn repeats had little detectable TaHsfA2b binding. As some functional TaHsp HSE sequences had one-nucleotide degeneracy, three synthetic sequences (GAAn2GTCn2GAA, TCCn2GCAn2TTC, and AGAAn2TTCT) were tested for TaHsfA2b binding activity. Either non-detectable or <10% of the TaHsp90.1E1 activity was observed in these synthetic oligonucleotides. These data indicate that single-nucleotide degeneracy in a perfect HSE sequence is allowed only in certain positions and three nGAAn inverted repeats are required for effective binding by HsfA. In addition, comparison of TaHsp17E1 and TaHsp17E2 binding activity indicates that the sequence flanking the GAA or TTC core motif also affects Hsf binding. The binding analysis together with transactivation assays implies that *TaHsp17* and *TaHsp90.1-A1* are likely to be the direct target genes of TaHsfA2b.

### TaHsp90.1E1 and TaHsp17E1 are functional heat shock elements and responsible for transactivation by TaHsfA2b and TaHsfC2a

**Fig. 9.**
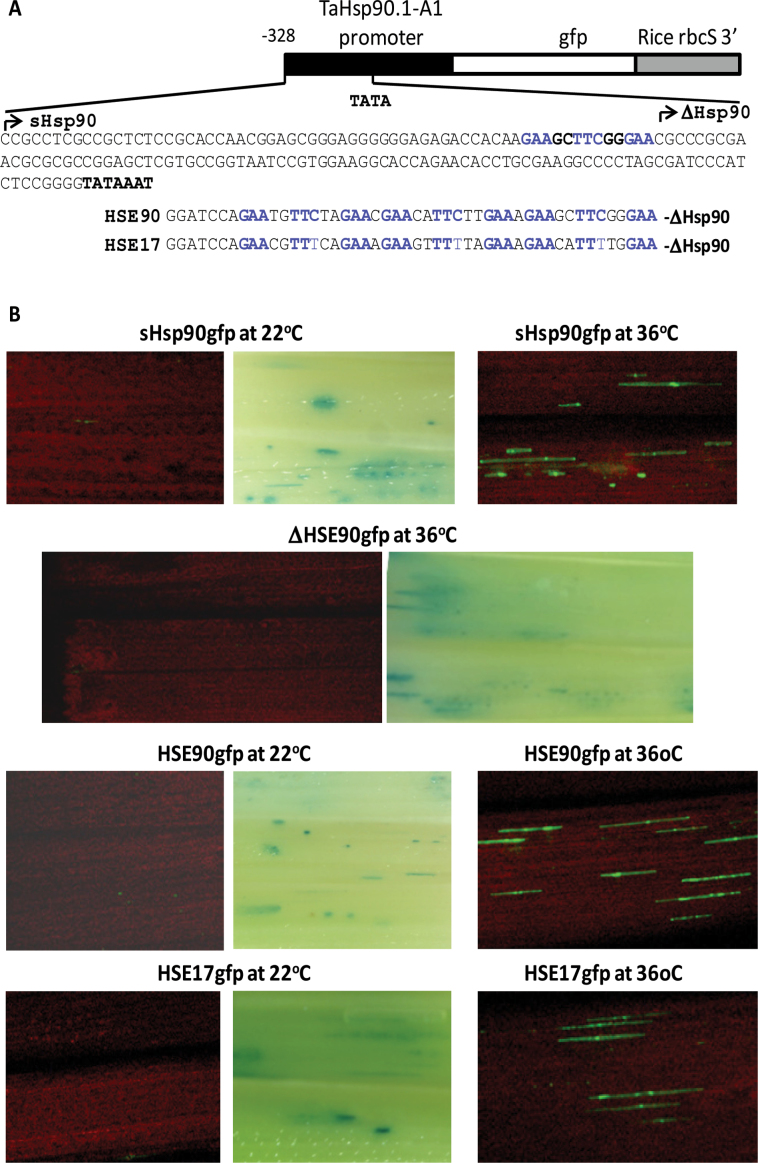
The heat-induced expression of *gfp* reporter genes driven by a truncated *TaHsp90.1-A1* promoter or a minimal promoter with an addition of TaHsp90.1E1 (**GAA**GC**TTC**GG**GAA**) or TaHsp17E1 (**GAA**CA**TTT**TG**GAA**). (A) Reporter gene constructs. A short *TaHsp90.1-A1* promoter (sHsp90) has a 328bp fragment upstream of the translational start codon, which contains the TaHsp90.1E1 element. The sequence upstream the TATA box of the *TaHsp90.1-A1* promoter is shown. The ΔHsp90 promoter starts immediately downstream of the TaHsp90.1E1 element. HSE90 and HSE17 constructs contain three TaHsp90.1E1 or TaHsp17E1 repeats, which are added immediately upstream of the ΔHsp90 promoter (a *TaHsp90.1-A1* minimal promoter). (B) Transient expression assays of reporter genes. The Ubi1GUS reporter gene was also co-introduced, and GUS foci are illustrated when *gfp* expression was essentially undetectable.

To investigate whether TaHsp90.1E1 (**GAA**GC**TTC**GG**GAA**) and TaHsp17E1 (**GAA**CA**TTT**TG**GAA**) are functional HSEs *in vivo*, *TaHsp90.1-A1* mutant promoters with truncation around the TaHsp90.1E1 region and *TaHsp90.1-A1* minimal promoters with an addition of TaHsp90.1E1 or TaHsp17E1 were tested (Fig. 9A). The *gfp* reporter gene driven by a short *TaHsp90.1-A1* promoter fragment (328bp upstream of its translation start codon) (sHsp90gfp) was strongly activated by heat treatment, while a deletion in TaHsp90.1E1 (ΔHSE90gfp) completely abolished its heat-inducible expression (Fig. 9B). Addition of TaHsp90.1E1 or TaHsp17E1 to ΔHSE90gfp (the minimal *TaHsp90.1-A1* promoter) restored its heat-inducible expression (Fig. 9B).

**Fig. 10.**
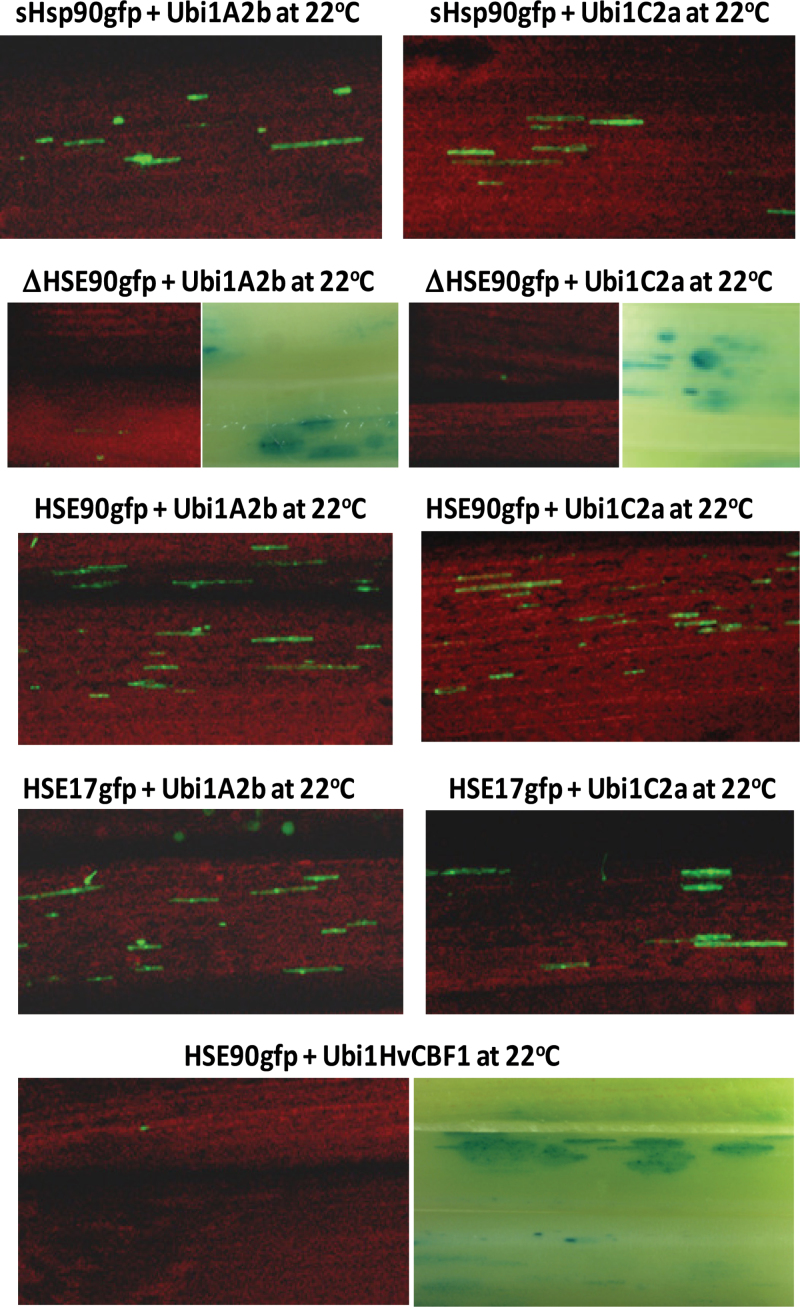
TaHsfA2b and TaHsfC2a transactivate the *gfp* reporter driven by the *TaHsp90.1-A1* minimal promoter containing TaHsp90.1E1 (HSE90) or TaHsp17E1 (HSE17). Constructs used are shown in Fig. 9. The Ubi1GUS reporter gene was also co-introduced, and GUS foci are illustrated when *gfp* expression was essentially undetectable. *Ubi1* promoter-driven HvCBF1, which is a transcriptional activator for cold-inducible genes, was used as a negative control.

With these *TaHsp90.1-A1* mutant promoter reporter genes, it was investigated whether TaHsp90.1E1 and TaHsp17E1 are responsible for transactivation by TaHsfA2b and TaHsfC2a. As shown in Fig. 10, expression of the *gfp* reporter genes driven by a sHsp90, HSE90, or HSE17 promoter was strongly activated by both *Ubi1* promoter-driven *TaHsfA2b* and *TaHsfC2a* effector genes without heat treatment. TaHsfA2b and TaHsfC2a were unable to activate the expression of a TaHsp90.1E1 deletion construct (ΔHSE90gfp) (Fig. 10), indicating that TaHsp90.1E1 and TaHsp17E1 are essential for transactivation by these two Hsf proteins. To confirm whether the transactivation of these reporter genes is specific to Hsf–HSE interaction, *Ubi1* promoter-driven barley CBF1 (Ubi1HvCBF1), which is a transcriptional activator of cold-inducible genes (Xue, 2002*b*), was also tested. The HSE90gfp construct was not transactivated by the Ubi1HvCBF1 effector gene (Fig. 10).

### TaHsfC2a contains a functional AHA motif that is responsible for its activation activity

**Fig. 11.**
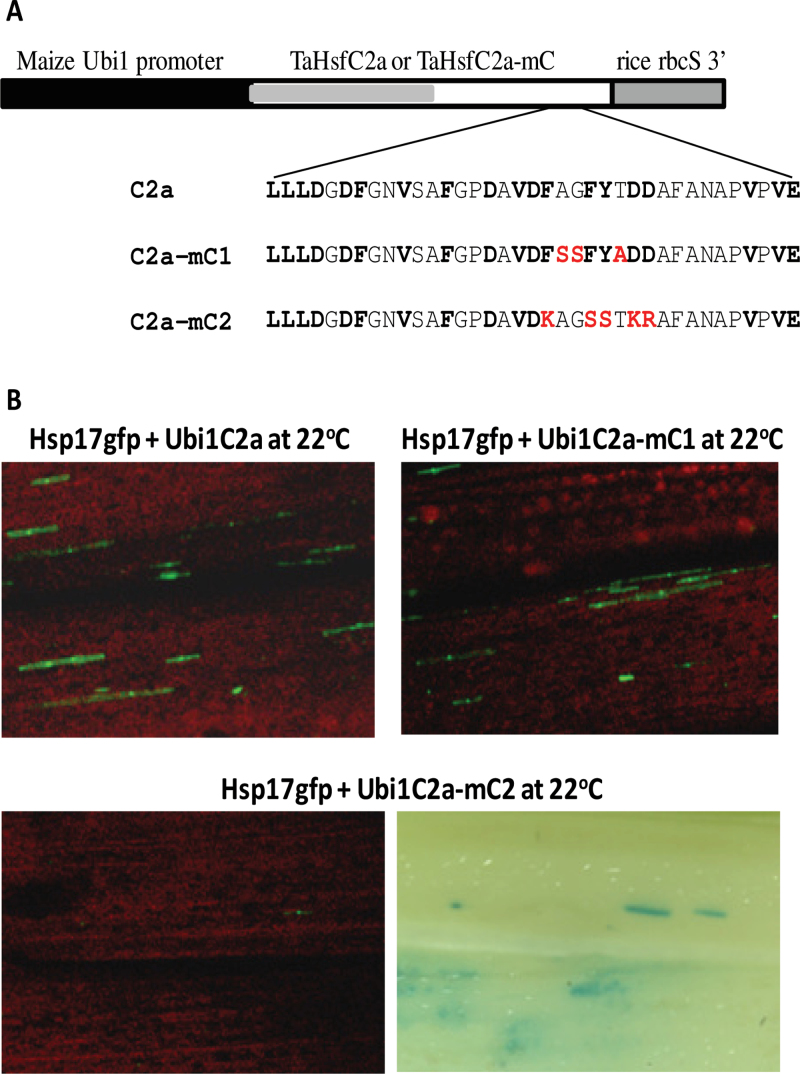
Loss of the transactivation activity of TaHsfC2a by mutation of hydrophobic and acidic residues in its C-terminal domain. (A) TaHsfC2a and its mutant constructs. The substituted amino residues in the mutants (C2a-mC1 and C2a-mC2) are in red. (B) Transactivation of the *TaHsp17* promoter-driven *gfp* reporter (Hsp17gfp) by TaHsfC2a and its mutants. The Ubi1GUS reporter gene was also co-introduced, and GUS foci are illustrated when *gfp* expression was essentially undetectable.

Transactivation of TaHsp90.1E1- and TaHsp17E1-containing promoters by TaHsfC2a is particularly interesting, as it is generally considered that HsfC class members do not contain a transcriptional activation domain. Therefore, it was investigated whether an AHA-like sequence (**LLLD**G**DF**GN**V**SA **F**GP**D**A**VDF**AG**FY**T**DD**AFANAP**V**P**VE**) present in TaHsfC2a is responsible for its transactivation activity. Two TaHsfC2a mutant constructs (C2a-mC1 and C2a-mC2) were made with mutation at the AHA-like region (Fig. 11A). The Ubi1C2a-mC2 construct containing a replacement of three aromatic residues (FxxFY) with KxxSS and two acidic residues (DD) with KR abolished its transactivation activity of the *TaHsp17* reporter (Hsp17gfp) (Fig. 11B), while the mutation in the non-AHA residues of this AHA-like motif (Ubi1C2a-mC1) retained its transactivation activity. This experiment demonstrated that this AHA-like motif in TaHsfC2a is responsible for its transactivation activity.

## Discussion

This study identified 56 Hsf genes from *T. aestivum*, which can be divided into three classes, A, B, and C. Although not all Hsf members have been identified in this study because the wheat genome sequence is still incomplete, analysis of the phylogentic tree of Hsf proteins from wheat and rice shows that the representatives of all rice subclasses have been identified from wheat (Fig. 1). Phylogenetic analysis showed that TaHsf proteins are more closely clustered with rice Hsfs. Similar to rice, the wheat Hsf family does not contain A9 and B3 subclasses as are present in *Arabidopsis*, but has C2 subclass members. Phylogenetic analysis also indicated strong support for separate B and C classes of Hsfs, but class A Hsf protein members formed at least three smaller clusters. Two of the class A clusters appear to be more similar to class C Hsf proteins than to the third class A cluster. This observation suggests that the A class is paraphyletic and that the C class is derived from a branch of the A class or that some subclasses of class A are really part of the C class. A similar observation was made by Scharf *et al.* (2012) when describing the phylogenetic relationships across Hsfs from nine plant species including both monocots and eudicots. They also noted a paraphyletic grouping of the A class Hsf members, whereas the B and C classes of Hsfs were monophyletic. It should also be noted that the branching patterns seen in the current phylogenetic tree figures may be influenced by incomplete data sets (missing wheat Hsfs). In comparison with *Arabidopsis* and tomato, monocot species (wheat and rice as well as *Brachypodium distachyon*) in general have fewer A1 members, and more A2 and C members per genome (Table 1; Scharf *et al.*, 2012). However, not much is known about the functional diversification of Hsfs in monocots to date.

In this study, the detailed expression profiles of individual TaHsf members in six important organs and heat responses were provided. The subclass A1 group members in wheat were found to be constitutively expressed at relatively high levels in all organs examined and were not up-regulated by heat stress, which is similar to *HsfA1* genes from other species such as *Arabidopsis* and rice (Hübel and Schöffl, 1994; Mittal *et al.*, 2009). However, there were many other constitutively expressed TaHsfA members (e.g. the A8 group, and some of the A2 and A6 group members) that were also expressed at relatively higher levels. A2 and A6 members were the main TaHsf members within class A that were up-regulated at a very high level during heat stress. These Hsf proteins are likely to be major transcription factors for up-regulation of Hsp genes during heat stress, as observed in wheat leaves and roots. Other class A genes that were up-regulated by heat were members of the A7 group and some A4 members, but they were expressed at a low level under heat stress, compared with the A2 and A6 groups. However, in terms of fold induction, heat up-regulation of *TaHsfA7* genes was very high. For example, the *TaHsfA7a* mRNA level was increased by 127-fold in the leaves at the 1.5h heat treatment (data not shown). In class B of the TaHsf family, the B1 and B2 groups were strongly up-regulated by heat; however, the expression levels of the B1 group gradually decreased after an initial strong induction. In class C, the C1 group were constitutively expressed at a high level in most organs. In young leaves, the expression levels of the C1 group were even higher than those of the A1 group. The C1 group members were generally down-regulated by heat stress, and their heat response pattern appears to be in the opposite direction to that of the heat-up-regulated A2 and A6 group genes. The C2 group members were predominantly expressed in the embryos and roots, and were also generally down-regulated by heat stress, except C2a and C2b that were up-regulated in the leaves by a short-term heat treatment. Interestingly, marked up-regulation of HsfC2 genes from rice by oxidative and heat stresses or a combination of these stresses has been reported (Mittal *et al*., 2009, 2012).

From analysis of TaHsf expression profiles, two interesting features are worth mentioning here. First, a number of Hsf members other than the A1 group were constitutively expressed at a level similar to or higher than the A1 group, presuming that they are generally present in an inactive form as is the case for HsfA1 proteins known in other species (Hahn *et al.*, 2011; Scharf *et al.*, 2012). Secondly, in contrast to what has been observed in sunflower, *Arabidopsis*, and rice (Almoguera *et al.*, 2002; Kotak *et al.*, 2007; Chauhan *et al.*, 2011*a*), no seed-specific Hsf members were identified in wheat. However, three A6 members (A6c, A6d, and A6e) and A5b were predominantly expressed in endosperm under non-stress conditions. In addition, four A2 members (A2b, A2c, A2e, and A2f) were also expressed in the endosperm of non-stressed plants at a relatively high level. This suggests that the endosperm is rich in TaHsfA proteins under non-stress conditions, with the assumption that there is no major difference at the translational level among TaHsfA subclasses. The endosperm is an important organ for wheat productivity. Some enzymes involved in starch synthesis in several crop species are known to be thermolabile, such as soluble starch synthase from wheat endosperm (Keeling *et al.*, 1993) and pea embryos (Denyer and Smith, 1992) and ADP-glucose pyrophosphorylase from maize endosperm (Greene and Hannah, 1998). Biochemical analysis has shown that the crude extract of soluble starch synthase from wheat endosperm loses its enzyme activity significantly with a 15min treatment at 30 °C, and a decrease in the enzyme activity is noticeable even at 25 °C with a 2h treatment (Keeling *et al.*, 1993). Wheat crops in Australia and other warm climate countries often encounter high temperatures above 30 °C at the grain-filling stage, which results in a significant reduction in grain yield and quality (Skylas *et al.*, 2002). The A6 and A2 Hsf genes that were highly expressed in the endosperm are likely to be important Hsf members for keeping the expression of Hsp genes at a high level. Indeed, four out of the five TaHsp genes examined were also expressed at a high level in the endosperm under non-stress conditions, while their mRNA levels in the vegetative organs were either not detectable or at a much lower level. It is tempting to speculate that this expression pattern of heat-inducible TaHsp genes may have an advantage in protecting this important organ against heat stress. Many Hsps are known to function as molecular chaperones and are capable of stabilizing thermolabile proteins against heat denaturation (Grover *et al.*, 2013; Waters, 2013). For example, a mitochondrial small Hsp can improve the electron transport activity of the mitochondrial NADH:ubiquinone oxidoreductase complex I in plants at high temperatures (Downs and Heckathorn, 1998). Similarly, a small Hsp from the chloroplast protects the chloroplast photosystem II electron transport activity from heat inactivation (Hechathorn *et al*., 1998).

Besides their role in wheat adaptation to heat stress, a number of TaHsf groups (A2, A3, A4, A6, B1, and C2) were up-regulated in the flag leaves and glumes of wheat at the grain-filling stage by drought stress. The up-regulation of these genes as well as the A8 and C1 groups was also seen in the shoots of salt-stressed plants. A similar salt up-regulation of these genes was observed in the roots, with the exception of the A8 and C2 groups. These data indicate that some of TaHsf members may have a regulatory role in wheat adaptation to drought and salt stress. Intriguingly, an examination of the expression levels of *TaHsp16.9*, *TaHsp17*, *TaHsp26.6*, and *TaHsp90.1* genes in drought- and salt-stressed wheat plants in the Affymetrix wheat genome array data sets (TA23 and E-MEXP-971) did not reveal up-regulation of these TaHsp genes by drought and salt stresses (data not shown). This suggests that drought- or salt-induced up-regulation of these TaHsfs has no impact on the expression of these TaHsp genes. Possible explanations for this observation include that TaHsf proteins are inactive, or that Hsp genes are in a strongly repressed state in these salt- and drought-stressed organs. Whether these TaHsfs are able to regulate the expression of other stress protection genes during salt and drought stresses requires future investigation. However, it has been shown that overexpression of some HsfA genes, such as *AtHsfA2* and *HaHsfA9*, can result not only in thermotolerance but also in salt and dehydration stress tolerance (Ogawa *et al.* 2007; Prieto-Dapena *et al.*, 2008), implying that some drought- and salt-inducible TaHsf members may have a similar role.

In this study, some functional HSEs present in the promoters of *TaHsp17*, *TaHsp26.6*, *TaHsp70d*, and *TaHsp90.1-A1* genes, which can be strongly bound by TaHsfA2b, were identified. Most of these functional HSEs in the promoters of these wheat Hsp genes contain imperfect HSE sequences, some of which had TaHsfA2b binding affinity even higher than that of the perfect HSE in the *TaHsp90.1-A1* promoter. It was shown that TaHsfA2b and TaHsfA4e were transcriptional activators and capable of activating the expression of *TaHsp17-* and *TaHsp90.1-A1* promoter-driven reporter genes in the leaves and roots. It appears that TaHsfA2b is a more potent transcriptional activator in transactivation of the *TaHsp17* promoter-driven reporter gene than TaHsfA4e. However, transactivation analysis showed that TaHsfB1b and TaHsfC1b had no ability to activate *TaHsp17* and *TaHsp90.1-A1* expression. As no direct evidence in previous studies has shown that B1 and C1 members are transcriptional activators of Hsp genes, it is likely that TaHsfB1 and TaHsfC1 proteins are not responsible for up-regulation of these Hsp genes in wheat, although *TaHsfB1* genes were up-regulated 5- to 10-fold in the leaves and roots at the first 1.5h heat treatment. In heat-stressed tomato, HsfB1 acts as a transcription co-activator, cooperating with class A Hsfs (Bharti *et al.*, 2004), while *Arabidopsis* HsfB1 functions as a repressor (Czarnecka-Verner *et al.*, 2004). However, rice HsfC1a and HsfC1b can potentially serve as transcriptional activators, as the transactivation activity of these two HsfC1 proteins has been observed in yeast cells by fusion with the Gal4 DNA-binding domain (Mittal *et al.*, 2011).

Promoter truncation and mutation analyses showed that the TaHsp90.1E1 (**GAA**GC**TTC**GG**GAA**) of the *TaHsp90.1-A1* promoter is the HSE responsible for its heat-induced expression by Hsf proteins. An atypical HSE identified in the *TaHsp17* promoter, TaHsp17E1 (**GAA**CA **TTT**TG**GAA**), is also a functional HSE *in vivo*. The addition of either TaHsp90.1E1 or TaHsp17E1 to a minimal promoter derived from the *TaHsp90.1-A1* promoter resulted in the heat-inducible expression and transactivation by TaHsfA2b or TaHsfC2a at 22 °C. These data implicate that the presence of either TaHsp90.1E1 or TaHsp17E1 is sufficient for heat-mediated expression through interaction with Hsf proteins.

Most interestingly, it was observed that TaHsfC2a acted as a transcriptional activator and was capable of transactivating the expression of the *TaHsp17* or *TaHsp90.1-A1* promoter-driven reporter gene. No previous studies to date have shown that a class C Hsf protein can transactivate the Hsp genes. The C-terminal end of TaHsfC2a contains a motif, **LLLD**G**DF**GN**V**SA**F**GP**D**A**VDF**AG**FY**T**DD **AFANAP**V**P**VE,** which resembles an AHA motif (Nover and Scharf, 1997; Kotak *et al.*, 2004). The mutation of some aromatic and acidic residues in this motif resulted in the abolishment of TaHsfC2a transactivation activity, suggesting that this AHA-like motif is responsible for its transcriptional activator property. TaHsfC2a was predominantly expressed in the seeds, particularly in the embryo, but was also heat inducible in the leaves with a short-term heat treatment (a 5-fold increase). Up-regulation of rice *HsfC2a* by a short-term heat treatment (10–30min) has also been shown in rice seedlings (Mittal *et al.*, 2009). An attempt was also made to investigate whether TaHsfC2a is capable of binding to the functional HSEs present in the TaHsp promoters *in vitro*, but the experiment failed technically due to a very low level of TaHsfC2a–CelD fusion protein produced in *Escherichia coli*. However, the transactivation of *TaHsp17* and *TaHsp90.1-A1* genes by TaHsfC2a appears to be through its specific interaction with TaHsp17E1 and TaHsp90.1E1 elements, as the promoter mutagenesis experiment demonstrated that the transactivation of *gfp* reporter genes by TaHsfC2a relied on the presence of either TaHsp90.1E1 or TaHsp17E1. Based on its expression pattern and ability to activate Hsp genes, TaHsfC2a probably plays a role in regulation of Hsp expression in the embryos and during early heat stress.

Another intriguing point raised by this study is that transactivation of Hsp genes by TaHsfA2b, TaHsfA4e, and TaHsfC2a does not require heat treatment, indicating that TaHsf proteins are functional at 22 °C. Presumably, they are able to form functional trimers under non-stress conditions. The question is why heat-inducible Hsp genes such as *TaHsp17* are not expressed in the leaves and roots of wheat under normal conditions, as some A2 (A2a, A2d, and A2f) and C2 (C2d and C2g) members were expressed in these organs at a level similar to or higher than that of TaHsfA1 members. It has been shown that some repressors (e.g. Hsp70 and Hsp90) bind to Hsf protein to keep them in an inactive monomer form (Kim and Schöffl, 2002; Yamada *et al.* 2007; Hahn *et al.*, 2011). At present, a model of Hsf activation derived from tomato plants is that HsfA1 is a master regulator of the heat response (Mishra *et al.*, 2002), and is in an inactive state through complexing with Hsp90 and Hsp70 (repressors) in non-stress conditions (Hahn *et al.*, 2011; Scharf *et al.*, 2012). In *Arabidopsis*, there is no single master Hsf, and three A1 members together serve as heat response-triggering factors (Liu *et al.*, 2011; Liu and Charng, 2012). Upon heat stress, Hsp90 and Hsp70 are released from HsfA1 to form a functional trimer, which subsequently activates expression of other HsfA members. However, constitutively expressed HsfA genes in wheat include some A2, A6, and A8 members in addition to A1 members. This implies that some repressors can keep all these constitutively expressed TaHsf transcriptional activators in an inactive form in wheat. An alternative hypothesis is that some repressors (e.g. transcriptional repressors) other than Hsp90 and Hsp70 may interfere with Hsf transcriptional activity at the Hsf protein and Hsp promoter assembly point in wheat to keep Hsp genes in a repressed state under non-stress conditions. These hypotheses suggest that Hsp gene activation by the Hsf proteins in wheat plant cells is likely to be dependent on the relative amounts of TaHsf proteins and their repressors. When TaHsf protein amounts are in a substantial excess compared with the amounts of their repressors such as Hsf overexpression in the transactivation assays or heat stress, Hsp genes become activated. This may also apply to the high level expression of some heat-inducible Hsp genes (e.g. *TaHsp17*, *TaHsp26.6*, *TaHsp70d*, and *TaHsp90.1A1*) in wheat endosperms and embryos under non-stress conditions.

It will be interesting to investigate whether genetic variability in Hsp levels and thermotolerance in wheat detected in early studies (Zivy, 1987; Krishnan *et al.*, 1989; Vierling and Nguyen, 1992; Skylas *et al.*, 2002) is contributed by differences in expression of some critical regulators—TaHsfs. Possibly, some TaHsf genes could be important quantitative trait loci for determining thermotolerance. A substantial amount of research is still required to unlock the mystery of a Hsf–Hsp regulation model and the molecular mechanisms of heat adaptation in this important crop species—wheat. This will be an important research issue to address in the future, to assist in ensuring an adequate food supply in the predicted global warming scenario.

## Supplementary data

Supplementary data are available at *JXB* online.

Figure S1. Deduced protein sequences of TaHsf genes.

Figure S2. Multiple sequence alignment of the DNA-binding domains of TaHsf proteins.

Figure S3. Multiple sequence alignment of HR-A core and HR-B regions of TaHsf proteins.

Figure S4. Neighbor–Joining phylogenetic tree of wheat Hsf proteins.

Figure S5. *TaHsp26.6* and *TaHsp70d* promoter sequences assembled from sequence databases.

Table S1. Real-time PCR primers of *T. aestivum* genes.

Table S2. A list of sequence IDs used for assembly of TaHsf genes.

Supplementary Data

## Abbreviations:

AHA: aromatic–hydrophobic–acidic residues
HSE: heat shock element
Hsf: heat shock factor
Hsp: heat shock protein
HR: heptad repeat.
