# Supplementary material for: The heat shock factor family from Triticum aestivum in response to heat and other major abiotic stresses and their role in regulation of heat shock protein genes
Source: J Exp Bot. 2013 Dec 9;65(2):539–57. doi: 10.1093/jxb/ert399 (PMC3904712; doi:10.1093/jxb/ert399)
Supplement: Supplementary Data [file supp_65_2_539__index.html]

The heat shock factor family from Triticum aestivum in response to heat and other major abiotic stresses and their role in regulation of heat shock protein genes — The heat shock factor family from Triticum aestivum in response to heat and other major abiotic stresses and their role in regulation of heat shock protein genes — Supplementary Data 

# The heat shock factor family from *Triticum aestivum* in response to heat and other major abiotic stresses and their role in regulation of heat shock protein genes

## Supplementary Data

Data files

**Files in this Data Supplement:**

- Supplementary Data - Supplementary Data
