# Supplementary material for: The heat shock factor family from Triticum aestivum in response to heat and other major abiotic stresses and their role in regulation of heat shock protein genes
Source: J Exp Bot. 2013 Dec 9;65(2):539–57. doi: 10.1093/jxb/ert399 (PMC3904712; doi:10.1093/jxb/ert399)
Supplement: Supplementary Data [file supp_ert399_jexbot105478_file001.pdf]

The heat shock factor family from *Triticum aestivum* in response to heat and other major abiotic stresses and their role in regulation of heat shock protein genes

Gang-Ping Xue, Shahab Sadat, Janneke Drenth and C Lynne McIntyre

Supplementary data

Supplementary Table S1. Real-time-PCR primers of *T. aestivum* genes

Gene	Forward primer	Reverse primer
TaHsfA1a	5′-AGCATTCCAGGATTCCCAGAT	5′-CCAGGCATTGCGAAATTCTC
TaHsfA1b	5′-AGCATTCCAGGATTCCCAGAT	5′-TCAGGACCAGGCATTGCATA
TaHsfA1c	5′-ACATGAGTGCTGTGCCTTCG	5′-GATCTGGGAATCCTGGAATACTGA
TaHsfA2a	5′-ATTTTCGTCAATGCCGTGTTTA	5′-GCATACAGCCCAATCCGACT
TaHsfA2b	5′-CATCTGATCTCGACGGCTCA	5′-GCTTCACAGGTACCGAGACCAT
TaHsfA2c	5′-CCCAAATAGTCCAACCCTCTCA	5′-CGACACTTGCATAGGCACCA
TaHsfA2d	5′-TCTAGTGGGTTGCTTGCGATT	5′-GCATACAGCCCAATCCGACT
TaHsfA2e	5′-CATCTGATCTCGACGCAGGTA	5′-GCTTCACAGGTACCGAGACCAT
TaHsfA2f	5′-CTGCTAGCTACTGTCGGTCTAACG	5′-CTGACAATGCCAATACACACGA
TaHsfA3a	5′-TTCCCGACAAGCATGAGAAA	5′-GGGTCACCGATAACGCACTT
TaHsfA3b	5′-TCCCGACAAGCCTGAGAGAT	5′-GGGTCGCCGATAACACACTT
TaHsfA4a	5′-CCAAGACCCTCCTGCTGATG	5′-CCTCCTGATGTGCATGGGATA
TaHsfA4b	5′-CCAAGACCCTCCTGCTGATG	5′-CCTGGTGTGCATGGGACA
TaHsfA4c	5′-TTCACCGGAGCTAGCAGAGC	5′-GTCGACTTCGGCCGTTGTAT
TaHsfA4d	5′-TCTTCACCGGAGATAGCAGAGTC	5′-GTCAACTTCGGCCATCGTCT
TaHsfA4e	5′-TCACCGGAGCTAGCGGAGT	5′-GTCGACTTCGGCCGTTGTAT
TaHsfA4f	5′-CCCAAGACCTTCCTGCTGAC	5′-CCAGGCTGCTCGGTAAGAAA
TaHsfA5a	5′-GGAGCACCACCATCAACGA	5′-CGACTGATGCCTTCGGGTTA
TaHsfA5b	5′-GCCATGGGCGTTAACCAG	5′-CTCTCGTGGCCTCAGTGCTT
TaHsfA6a	5′-ACGACTTCTGGGAGGAGCTG	5′-TCCTTTCCAGCTCTACCATCTCA
TaHsfA6b	5′-ACGACTTCTGGGAGGAGCTG	5′-CCTCCTCTCCAGCTCTAGCATC
TaHsfA6c	5′-AGCTGGACATCAACAGCGATT	5′-ACAGCCCAGACCCGTCTTC
TaHsfA6d	5′-GGTGGCAACATGACTGGCTA	5′-CCCAGAGGTGCCATCAACA
TaHsfA6e	5′-GATGATTTTGGAGACGGAGACG	5′-ACTGCTGAGCCAGTTCATCG
TaHsfA7a	5′-GATCGACTCCTTTGCCTGCT	5′-AGTGATCATGATGAATTGTGCTGA
TaHsfA7b	5′-AGGACCTGATGCAGCAGATAGAC	5′-CTAGTGAATTGTGCCGAGCTGA
TaHsfA8a	5′-AGGTGGTGGTCTAGGAGCACAT	5′-CTCTGAGCACAGATGCCGTTT
TaHsfA8b	5′-AGGTGGTGGTCTAGGAGCACAT	5′-GCATTGCTATACGACACTCTCACAA
TaHsfA8c	5′-AGGTGGTGGTCTAGGAGCACAT	5′-CACTCTGAGCACAACTCCTGCTT
TaHsfB1a	5′-GACTCCCCAGTCCTCTAAAACCA	5′-GGAACGCGACGTTTACGC
TaHsfB1b	5′-CGACTCCCCAGTCCTCGAA	5′-GGAACGCGACGTTTACGC
TaHsfB1c	5′-ACGACTCCCCAGTCCTCCA	5′-GGAACGCGACGTTTACGC
TaHsfB2a	5′-GTCGGGAATTGCTCTGGGT	5′-AAGCGCGGCTCTTCTCCT
TaHsfB2b	5′-CTAGGCTCTTCGGCGTCTG	5′-ATCGGCTCAGGCTTCACCT
TaHsfB2c	5′-GGCGAGGAGCTTCCTAACG	5′-CCACGACTGCCGATCCTC
TaHsfB2d	5′-CGTCTCCATCGGGCTGAA	5′-CGTCGCCGTCTTCTTCCTC
TaHsfB2e	5′-GCGAGGAGCTTCCTAACAGC	5′-CCACGACTGCCGATCCTC
TaHsfB4a	5′-AGTTCGCCAACGAGTTCTTCA	5′-GGCACCATTGAAGGCAGAG
TaHsfB4b	5′-AGTTCGCCAACGAGTTCTTCA	5′-CTTCCTCCGGTGGATCTCG
TaHsfB4c	5′-GCACTTCCCGCTGTTCCA	5′-CCCATCCTCACCCATGAACT
TaHsfC1a	5′-AGCAGCATGGGCATAACTGA	5′-CGCGCCGTGTTTAGAAGAA
TaHsfC1b	5′-GTGCCGTTCCCCTTCTCTAA	5′-TCTATGAACTCTCGACTCCCAAGG
TaHsfC1c	5′-GTGCCGTTCCCCTTCTGTAA	5′-ATCTATCAACTCTCGACTCCCAAGG
TaHsfc1d	5′-CGTGCCGTTCCCCTTATCTA	5′-ATCAACTCTCGGCTCCCAAG
TaHsfC2a	5′-GCGACTTCGGCAACGTATC	5′-GCGTCGTCGGTGTAGAACC
TaHsfc2b	5′-GTGAGAAGAGGGCGAGGATG	5′-GCGTCGTCGGTGTAGAACC
TaHsfC2c	5′-GCCCAGCGTGGAATTGTACT	5′-CAGTAGCCGCTGTTGACGTG
TaHsfc2d	5′-CGTACGCTTTCCCTGTGGA	5′-TGTGTCGGTGAAACACCCTCT
TaHsfC2e	5′-GGCGGCTACTGAGTTGGTTAAG	5′-CCACGTCGTCTAAAAAGCCAAA
TahsfC2f	5′-CGACGGGTTCAGCGATGT	5′-AGCTACCGTCCACGGTGAAA
TaHsfC2g	5′-GCCGTACGCTTTCCGTGT	5′-TGTGTCGGTGAAACACCCTCT
TaHsp16.9	5′-GTGTGCGAGTTCTTGCGAGTC	5′-CTGTTGGCGATTAGCCGATTA
TaHsp17	5′-GTGCGAGCTCTGCCACCT	5′-GTACAGATGCCTCGCCACAC
TaHsp26.6	5′-GATGAGTTTGTGCGAGACTCTGTAC	5′-GAACGGCACCCTACGTCTACA
TaHsp70d	5′-CGCGCTACTTGATGGGTTC	5′-GAGCGGCATCCAAAGGAC
TaHsp90.1-A1	5′-GTCTCATTTCCTTGCAACCAGG	5′-CTAGACACGCAGCGACCAAC
TaRP15	5′-GCACACGTGCTTTGCAGATAAG	5′-GCCCTCAAGCTCAACCATAACT
TaRPII36	5′-ACGTATTAACCAAGAACTCATGGAGAC	5′-TCAAATACTTTTGTAGGGCTGCTCTC

Supplementary Table S2. Sequence IDs for assembly of TaHsf genes

Gene	Full or Partial (C or N) ORF	GenBank Accession # (Sequences with IDs in bold were isolated from this study)	Wheat genomic sequence ID #
TaHsfA1a	Full	**KF208541**, HX141174, CJ936898, HX141200, CJ696176, CJ640745, CJ924783, CJ589968, CJ532366, CJ590095	GINDECP04B1KJM, GG7JGRH08HNBHB, GKWV9KP06H9Z6E, GJOJZ1301CDXFR, F3THAEN01BMZ29, GBO9LGD02F9E4V, GIPCY5M01COCCU, GGSKSHR02GTBZX, GKWX1DQ02C4UJJ, GJMPFVZ02IY8Z7, GH8MDFQ04BY7FK, F2S221K01AFO90, GMB8IBH02HZGJ4, GJVZZDM01DW3QN, GJVZZDM01DOIZ7, GL5C4KS01ANK6D, GIABLPU04D4P10, GJVZZDM01CHGVE, F3ZQ05F01EYF7B
TaHsfA1b	Full	**KF208542**, HX130064, CJ642331, CA608191, CA604188, CD896030, CJ534010, DR735777, CN010987, CD893842	GDH717U01CDZUT, GJOJZ1302F3R2D, GMC3LKS01DCMWU, GJ20S3U02JB2I5, GIOY55M01DPXRT, GMC3LKS01DBS5D, GKTGI0Q01CGPEA, GHY43EF05FR0HA, GKW2O1Y01BW5AI
TaHsfA1c	Almost full	**KF208543**, BE490218, BQ161207	GCRXJAC01EVMPF, GJZO9OG02GK84R, GIPCY5M01A8VK5, GIB2VHM02GLX8B, GKU3SMK02ER7S1, GKU3SMK03DFEM4, GGWH0HJ01EZBN4, GH96DCX02FVQW4, GGWF2QU01EX6CT
TaHsfA2a	Full	BJ239127, BJ233478	GGE9MRJ02GAMZX, GGQTHUK02I8DNS, GJMPFVZ01BYLE6, GKW2OSF02JU9JT, GKNVW9M02GRL3E, GKTGGAA01D93D6, F3QGDP101A15OD, GJOJZ1301A6W9I, F3QGDP102H81WR, GCMVUI302IFNWK, GINEZBA01DEOHD, F05ICCK01EX7VT , GHY43EF01EJ6O8, GCMIFJO02JAR7C, GI0FJWC02HYGWI, GMB8IBH01CS80K, GGWE0EB01AZP7R GIPCY5M01BLEBQ, F27137E02ID9UE, GKY3Q6202F8STH, GG7JGRH05GR6B3, GIYOKMP01C6CJ2, GDH717U01BM7NP, F3ODV4H02JBU6V, GH95CX402I00IY , GJVZXJB02JWYL7
TaHsfA2b	Full	**KF208544**, BI479783, BJ304701, BJ232988, BJ310504 , BJ238629, CJ524164, CJ632700, CJ524152	GJBO7DZ01A3K55, GGE9MRJ01C5IQE, GJZO9OG01EIV3Q, F6VABRJ08JF6ZH, F3TTDBY02IOC81, F4UURYP02HQU8U, GKYPTCQ07HYFVW, GKHV1DN01BI33X
TaHsfA2c	Full	CD939626, CJ649060,CJ608117 , CJ608116, CJ712793, CJ712794, CJ608125, CJ682497, CJ575801, CJ540832, CD934428	GGWF2QU02FW489, GL5C4KS01DIBO4, GG1XLLD02BSBA9, F1XBOI002JTODA , GKNV0OF02IXZMY , F2HXLMV01EBESZ , GJP3OI101EDXF6
TaHsfA2d	Partial(C)	BJ245724, BJ251614	GINEZBA03A4VR2, F019BF102H70K6,GH8MDFQ03D2B5H, GKL6WN402HZDT3, GKK8JBB02FG5DR, GKK8JBB02FG5DR, GKU3SMK08HOMXE, GIV7BON03GVY9O, GJVZZDM02FL00O, F2HXLMV02FQ6V8, GIV7BON03GJO52, GGSKSHR02F02DC , GJ20ROV01EHZGX, GKF76DR02G0PS0, GJVZZDM02HZS93 , GKF77RY02I2YNQ , GJ6VB8N01CJ94W, GBD1UXH01BQF21
TaHsfA2e	Partial(C)	BJ249032, BJ265879, BJ260181, CA650042, BJ255282, CA634016	GJ6VE9I02G38LM,F2M8N5A02GYAXV, F33GM1K01EEOFS, F4WYWDN01BJQAE, GKL6WN401D5GGU, F676MBK01DVJLT, F0YKN2Z03CZIKK
TaHsfA2f	Partial(C)	DR736601, HX185202, HX185222 , BE423936, CK205954	GSKSHR02GH0BQ, GKWV9KP01DRQ0S, GHY6XPR04EJ9DI, GJVZXJB02GT9HS, GEDSYXS02IEQSH, GINDECP06F8PI8,
TaHsfA2g	Partial (N)	BE427506	F2HXLMV01EVDL, F6PXRVY02GUXP6, GKU1Y2K01AMXP0, GKJH3DB05IPY7X
TaHsfA2h	Full	**KF208545**, CA733194, CA732800	GBD1UXH01CIR5P
TaHsfA2i	Partial (N)	AJ603615	GKY3N4D02GMRQQ, GKY3N4D02I6GMV, F4J2DG101AVOHL
TaHsfA3a	Full	DR740109, CJ909568, CJ897735, BJ268638, BJ273582	GJBO7DZ02JIS8K,GG7JLI305GFMBW,GG7HVD104C4QF4, GKF76DR01EC6ZS,GG7HVD106IOBM4, GHIK20O02JMASG, F5ZM8CW01EDEU5, GGWH0HJ01AFGYK, GKJD2EX02I2XKZ, GD9UPD202FOXJX, GI0FJWC01BBHYH, GI0FJWC01ER05V,GI0FJWC01DX4V3
TaHsfA3b	Partial(C)	CJ873489, CJ885251	GG7JLI308F57DG, GI0FJWC01C5ICX
TaHsfA4a	Full	FJ790791, JQ801451, CJ553148,CD873225, DR740033, CJ660695, HX015148, HX015165, HX015166, CJ913322	GKNV0ZV02HGOOH , GD37MD302IDLG0 , F019BF101B8ED9, GKWX1DQ06HUJXW, GG1VXPK06HIC2Y, GJVZZDM01EGUZC, GKL6WMX02G2Z5Y, GG873SR01BXU9A , GH96DCX02IG7VX
TaHsfA4b	full	FN564426	F4UURYP01EEEL7
taHsfA4c	Full	**KF208546**, CJ883426, CJ890154, CJ878347, CJ901458, CJ949200, CJ949504, CJ961415, CJ961106	GBO9LGD02IZSO5
TaHsfA4d	full	CV758782	GIPCY5M02ICM4L, GGWF2QU02IEHPO, GKJDY9A02I1YGW, GKL6WMX01BA7AY, F3ODV4H02HUUGL, F6PK5LH01AK7HN,GKJGB6C06GPTC4, GIOY55M01C3XME, GKNVW9M01AV19H, GHY43EF08GWSSJ, GKJD2EX01BS8Z8, GI0FJWC01BYFU7, GKU1Y2K06J2YY9, GDHUJ9D01EG0KP, F1XNFFR02HHT47, GGWE0EB01EOABQ , GCRXJAC01EHZZ9, GCRXJAC01DUS29, GCRXJAC02IVB1M, GKU3SMK08GDXAO, GKU3SMK06IXPCW
TaHsfA4e	full	**KF208547**, CV762533, BJ229131, CV766704	GJ20ROV02HQ8HC, GKW2O1Y02GB3XI, F4ICKYS01DHE36, F676MBK02I9XY3, GKNVW9M02JRO69, GFAS8M201B7BQ6, GJVZZDM01DG27F, GKVDJ3A02GAF3E, GKY3Q2G02IU2JR, GKY3Q2G02I8BWZ , GIPCY5M01CF41Q, F6CVSS101BN260, F4W8D9R01B7JBD

TaHsfA4f	Partial(C)	CJ514096 , HX112929, CJ589903, BJ219646	GKL6WMX02IMIHQ, GK0RIMC02JAW7P, F01XQWW01A44RA, GGQO11T02GD0FH, GJVZXJB02IKYTR, GHGGQMV01AGRAD, GGWE0EB01B2QC8
TaHsfA5a	full	AL812570, CD871372, AL814343, CD871373	GJZOW2C02FUH2K, GJ20ROV02GO7J7, GKJD2EX01B4SV8, GINDECP01AXTHE, GKS5NH307IIPQ1, GKS5NH307HOIXN, GJ20ROV02GO7J7, F33GM1K01DVZ1N, GKJD2EX01CHYYM, F6CI9PH01C0ZDK, GKWX1DQ06JA16S, GJ20S3U01C2RZF, F5V704K02IYW1N
TaHsfA5b	full	CJ655373, CJ945724, CJ547566	GGWF2QU02HWTNK, GCRXJAC02FK7AE,GCRXJAC02GIKHA , GKTGI0Q02GP78T, GINEZBA03DJS0A, GINEZBA03BMVO2, GKL6WN401EKURB, GKL6WMX01EAB7X, GKU3SMK02DPK1W, F4JP4XR01BXMQ0, F5BLKSV01CNAD3, GKK8JBB01ASAXH
TaHsfA6a	Full	CD884036, CD881399, CV768109, CD882214, CD881400	GKJD2EX02JCPX7 , GI0FJWC02FZR4X, F4ICKYS01DBJLZ , GIOY55M02JCOM7, GKTGI0Q02G29O6, GMC3LKS02HFFF9, F4J2DG102GDLKH, F51T30G01EIT8A, F6JU48R01COH0Q
TaHsfA6b	Partial(C)	CJ555209, CJ662666,	GJ6U61T06HIA86, GJZO9OG01BN95F, GIOY55M01D93AA, F2SQ5YK02G9XUG, F03ZQ2H01CF0UN, GIPCY5M01CMUHI, GJMPFVZ02G8AHR, GKYNZJQ03EPN70, F2SQ5YK02F1GI9
TaHsfA6c	Partial(C)	HX033482, CJ572425, CJ679235, HX033494	F5OR3XZ01A3F7K, F4ICKYS01AEX8C, GKWX1DQ08H978B, GKTGI2C02HEDLL , GG7HVD105IMZEB
TaHsfA6d	Partial(C)	HX087760, HX087784, HX039319, GD186958, AJ602104, AJ604346	F019BF101C2DNJ, F5ZM8CW01BOTVK , GDHUJ9D02HYUGS, GKS5NH306JFFZ5, GKS5NH306HU6A1 , F4UURYP01DJHZI, GH96DCX02FON8G, GCRXJAC02G4CB7 , GJ6VE9I01BMFR6, GKU3SMK03DF65C
TaHsfA6e	Full	**KF208548**, AJ602330, HX034191, HX031605, HX031606, HX034202, HX011232, HX011220, CA595102, HX035147, HX031620	F27137E02HZD3T, F1XNFFR02I26UK, GKY3Q2G01BVLSA, GL5C2CV02GJQPT, GKU3SMK02C382N, GJP3OI102HAQNT, GJVZZDM02GUKKU, F4W8D9R02HAV0V, GKY3Q2G02IEXC4 , GMEN2LX02ICYY7 , GMEN2LX02FGHUQ
TaHsfA6f	Partial (N)	CN009110	F5V704K01A7CE6, GINEZBA03A6DHE
TaHsfA7a	Full	AJ601747, AJ603017, GT629372, GT629491	GKJH3DB03C6WJC, GGQTHUK01EN8LF, GCT78DC01D8UEZ, GKY3Q2G01CJFUW, GKHV1DN02F3G3X, GIB2VHM01EHMLI, GKW2OSF02JOKKP, GG873SR01E1MAC
TaHsfA7b	Full	AJ601565, AJ602329 AJ602992	F4H0DWW01DGKPO, GIB60WD05IZMCB, GJIP2BT01D2OBA, GJMPFVZ02GH1JQ, GIB60WD08IOC56,GGWE0EB02HQ3FY, GJZO2FP01CD4DW, GIB60WD07JT4IZ, GDH717U02H5H7P
TaHsfA8a	Full	CJ867391, CJ691879, CJ854848	GMB8IBH02H2GVG, GJP3QJH02F49M2, GCMIFJO02IN4IR, GMEN40301E5QXT, GHJ5B9401EX1E3, GJVZXJB02G7QP5, GD9UPD202IDCA1, F1XNFFR02JXHFE, GKNVW9M02G3CIB, GL5C4KS01DL2BK, GGQTHUK02HIOPJ, GGWFHOC01CA3X1, GKHV1DN02ILNHK, GGWFHOC01DCI50, GMB8J2D01ES45A, F62S5NF02I2EOF, F6JU48R01DL3TQ, GGWE0EB02G30OL, F1XBOI001B99BG, GGWFHOC02F47U2, GKU3SMK04EE268 , GDHUJ9D02JP4CM, GJBO7DZ01DB3KM , GKNVW9M01B3GES, F7IO22A02GFPAU, GEDH1DC01CQO8Y, GL5C4KS02JE47U, F1XNFFR01EQD30
TaHsfA8b	Partial(C)	CJ710626, CJ605460	GKL6WN402G6RBW, GKJH3DB02D2NF0
TaHsfA8c	Partial(C)	BM137452, BJ276234	F3TTDBY01C0ZSH, GL5C4KS02JQLUO
TaHsfB1a	Full	HX144929, CD881419, HX131473, CA596291, BE499216, HX155424, HX144955, HX155396, CN008139, BF484149, BQ169833, HX131473, BQ483077, BQ170572	GL5C4KS02JT9AB, GDEB4RJ02G5ZLQ , GK0RKIQ02HAO22, GJP3QJH01AJGE8, GKKCC0T01DRG8B, GKKCC0T01A84AW , GHJ5B9401EXUME, GCT78DC01B00YF, F3MO43G02GKF1E
TaHsfB1b	full	**KF208549**, CD920551, HX197208, HX165694, HX060305, CN011493, CD920552	F6JU48R01EHQ32, GGWH0HJ01BY7RS, F5V704K02JAOPL , GJBO7DZ01CH5QE, GKS7ENJ06HO4I9, GKU3SMK07I08ON, GCES68D02IO5JT , F05ICCK01E1TEJ
TaHsfB1c	Full	HX146375, HX146398	GIABLPU01CCTSQ, GB58NSO02FM6Y8, GD9UPD202G4OWN, GIB60WD01DY708, GHILVEO02HJW5L, GINDECP02B2USY, GINDECP01BR09Z, GAOC5IM01CLUNR, F03ZQ2H01ELLOL
TaHsfB2a	full	JQ771755 (= TaHsf3, Zhang et al., 2013)	GJ6VE9I02HXQIY, GHILVEO02IRXMV, GBO9LGD02HKM3B, GKKB9MV02J4G7Y, F62GJYH02H1W46, GCMVUI302IISY5, F1XNFFR02GQXCD
TaHsfB2b	Full	AK331145	GHJ5B9401CIHSQ, GHGGQMV01BPV8U, GJ6VE9I02HXQIY, GHILVEO02IRXMV, GBO9LGD02HKM3B, GKKB9MV02J4G7Y, F62GJYH02H1W46, GCMVUI302IISY5, F1XNFFR02GQXCD
TaHsfB2c	Full	AK331030, CA741191, AL821795, CK208429	GHJ5B9401AU33O, GKNVW9M01BIJ59, GKJD2EX02HBL3W, GKJD2EX02HS8EC , GG873SR02HXPXE

TaHsfB2d	Full	AK331994, CD894087, HX164079, CV781806, AL819671, HX164104	GJ20ROV02F317M, F6PXRVY02HZ8X0, F4J2DG101DH024, GIPCY5M01DVPI4 , GDS1A9H02I8XJ6, GGSJHGC01BJ6S5, GHJ5B9402GR0DQ, GEFGSVT01D8LSQ, F3MO43G01DUJVB, GHIK20O01B2CCA
TaHsfB2e	full	CV781279, CV762050, BQ237733	GJVZT6401BAYOT, F5ZM8CW02H6D5O, GGWH0HJ01CHPBT, GJMPFVZ01BIVBK, GGSKSHR01A8EWV
TaHsfB4a	full	**KF208550,** BU100082	GJMPFVZ02IZYT8
TaHsfB4b	Partial (N)	**KF208551**, AL818825	GHGGQMV02J5IUH, GIABLPU07GI21H, GGWFSIE01BOQEB,GG7JGRH02DCBCE, GJMPFVZ02IM2BX,GG7HVD108H5QYC, F4WYWDN01BY6AH , GGWFHOC01DVZC6, GDH717U01D43DV, GL5C4KS02HR8CS
TaHsfB4c	Partial (N)	BU100475	GCI7VBT02HDM1Z , GKY3Q6202I18KA, GJZO2FP02GKZ11, F33GM1K01D5701, GKVDNZY01CJVEC
TaHsfC1a	full	AK335755	GKS7ENJ07IBCE0, GIB60WD01BNAKM, GL5C2CV02IRV93, GKVDNZY01COU4J, GKVDJ3A01C5XA8, GKKB9MV02IRIIN, GMEN2LX01DOW38, GDTEVZE02IURUP, GG873SR02JMTDH, GGWH0HJ02H10NT
TaHsfC1b	Full	**KF208552**, CK208279, CD918044, CJ536864, AL816629, CJ645117	GKVDNAN02JDV1Z, F4JP4XR01BVA0X , GG1VXPK02EBYR0
TaHsfC1c	full	BE418746, BM134558, BQ161673	F5OR3XZ02IEGXP, F3QGDP102IPWET, GKKCC0T01D2ZIV, GH95CX401C16SJ,GHJ5B9402HNT5W
TaHsfC1d	full	CD924855	GGWF2QU01ECD6K, GKKB9MV01DCFIS, GEDSYXS01CYOZR, GCIUHBD01BLESV, GJVZXJB01DT8NF, GINEZBA04ESK83, GGQTHUK02IYGX7, GMEN2LX02JGQ7D, GKYNZJQ08F1J11
TaHsfC1e	full	**KF208553**	GDHUJ9D01A851H, GB58NSO02F4KXC, GD95LZB01AX3OJ, F4ICKYS01CJK1Q, GJZO2FP01A2X5B
TaHsfC2a	full	**KF234653**, DY741996, CD925960, BJ290804	GG873SR02HI0NH, GMB8IBH02HGIV9, F2NKJR101ECPA3, GGQTHUK01D98N2, GGQTHUK01AS3OJ, GJP3OI102JSAUN
TaHsfC2b	full	CV766170, BE488911, CD868305, CD868306, CD924507, BE426590, CA701457, GH728920	GG1XLLD03BOZNQ, F4W8D9R01C3EQG
TaHsfC2c	almost full	CD919573, CD919574	
TaHsfC2d	Full	AK336172, HX038167, HX014259, HX019123, CJ627951, CK196713, CJ519157, CJ519166, BJ296892, BJ289994, HX038183, HX014272, HX019131, CJ728027, CJ624243, BQ752863, CD909725	GKL6WMX02FP9WJ,GGQTHUK02FK3LT, GH99TNM04C5CE0, GKS7ENJ01CHNJB, GKY3Q6202G2DXL, GINDECP01AS9K5, GKHV1DN02IFNQX, F62GJYH02JT6GW, GINEZBA03D3Z4S, F62S5NF02FW53V, GKNV0ZV01BY62T, GD37MD301B28YY, GK0RIMC02I4LJ1
TaHsfC2e	full	BE470775, BE405149	GKJGB6C05GCC0Q, GGWGGLX01C3M41, GJ6VB8N02GDWLH, F2S221K01B48SI, GMEN40302JJLCM, GGE9MRJ01C3O34, GG7JLI306GKM5A, F2NKJR101BY4B7, GKS7ENJ04AY030, GDTEVZE02HYP6F,GJOJZ1301AQDET, GJOJZ1301DSRZV, GG1XLLD06G7AA4
TaHsfC2f	Full	**KF208554**, HX188176, HX168190, HX168219, HX188203	GINDECP01AS9K5>GH99TNM04C5CE0>GH99TNM04C5CE0
TaHsfC2g	full	BE471094, CD373418	GKKCCJJ01DZHE6, GH99TNM07IKQLH

TaHsfA1a
MDGGVAAVASAAAAAAAASSTVTTAVAPPGAGAGAGAPPPFLMKTYDMVDDPATDAVVSWGPANNSFIVWNTPEFARDLLPKYFKHNNFSSFVRQLNTYGFRKVDPDKW
EFANEGFLRGQKHLLKTINRRKPSHANNQVQVPQQQQHHQQQHQQQPQLQNAPMPSCVEVGKFGMEEEIEMLKRDKNVLMQELVRLRQQQQTTDHQLQTLGKRLHGMEQ
RQQQMMSFLAKAMQSPGFLAQFVQQNENSKRRIVAANKKRRLPKQDDGLNPESALLDGQIIKYQPMINEAAKAMLRKILQQDTSPHRFESMGNSDNLLLENCMPSAQTF
DSSSSTRNSAVTLAEVPGNSGMPYMPTSSGLSAICSSSTPPEMQCPVLDSNSSTQLPTQLPNMSAASSIPKAMTPGLSDISIPGFPDLHDLITEDAINIPVENFAMPGP
ECIFPLPDEGSDDSVPMDPIDTDETDDTQKLPGIIDSFWEQFLCASPLSIDNDEVDSGLLDTREAQEENGWTRTENLANLTEQMGLLSSNHRG

TaHsfA1b
MDGGVAAVAAAAAAASTVTTAVAPPGAAGAGAGAPPPFLMKTYDMVDDPATDAVVSWGPANNSFIVWNTPEFARDLLPKYFKHNNFSSFVRQLNTYGFRKVDPDKWEFANE
GFLRGQKHLLKTINRRKPSHANNQVQVPQQQPQQQHQQQPQLQNAPMPSCVEVGKFGMEEEIEMLKRDKNVLMQELVRLRQQQQTTDHQLQTLGKRLHGMEQRQQQMMS
FLAKAMQSPGFLAQFVQQNENSKRRIVAANKKRRLPKQDDGLNSESALLDGQIIKYQPMINEAAKAMLRKILQQDTSPHRFESMGNSDNLLLENCMPSAQTFDSSSSTR
NSAVTLAEVPGNSGMPYMPTSSGLSAICSSSTPPEMQCPVLDSNSSTQLPTQLPNMSAASSIPKAMTPGLSDISIPGFPDLHDLITEDAINIPVENYAMPGPECIFPLP
DEGSDDSVPMDPIDTDETDDTQKLPGIIDSFWEQFLCASPLSIDNDEVDSGLLDTREAQEENGWTRTENLANLTEQMGLLSSNHRG

TaHsfA1c
AAAAASTVTTAVAPPGAAGAGAGAPPPFLMKTYDMVDDPATDAVVSWGPANNSFIVWNTPEFARDLLPKYFKHNNFSSFVRQLNTYGFRKVDPDKWEFANEGFLRGQKH
LLKTINRRKPSHANNQVQVQQQQPQQQQQQQPQLQNAPMPSCVEVGKFGMEEEIEMLKRDKNVLMQELVRLRQQQQTTDHQLQTLGKRLHGMEQRQQQMMSFLAKAMQ
SPGFLAQFVQQNENSKRRIVAANKKRRLPKQDDGLNSESALLDGQIIKYQPMINEAAKAMLRKILQQDTSPHRFESMANSDNLLLENCMPSAQTFDSSSSTRNSAVTLA
EVPGNSGMPYMPTSSGLSAICSSSSPPEMQRPVLDTNSSTQLPNMSAVPSVPKAMTPGLSDISIPGFPDLHDLITEDAINIPVENFTMPGPECIFPLPDEGSDDSVPMD
PIDTDETDDTQKLPGIIDSFWEQFLCASPLSIDNDEVDSGLLDTRGAQEENGWTRTENLANLTEQMGLLSSNHRG

TaHsfA2a
MSHRMMMSPVKVEGRRCPDPDPAAAAGGGAPRPMDGLGDAGPTPFLAKTYDMVDDPATDAVVSWTATSNSFVVWDPHLFATLLLPSYFKHSNFSSFVRQLNTYGFRKVD
PDRWEFANEGFLRGQRHLLRNIKRRKPTHGSQNQQSLGSYLEVGNFGHDVEIDHLKRDRQLLMAEVVKLRQEQQNTRSDLQAMEKRLQGTEQKQQQMMSFLARVMQNPM
FIHHLLSQSEMRKELEDAISNKRRRRIDQGPEAVDSMGTGSTLEQGSQVMFEQQEPVDSLVNGVISDLESSSVDTKGAEVQQSVASSRSEQLRGRPSGELNDDFWEDLL
HEGGLGEEASNLVVPDDMNLLAEKLD

TaHsfA2b
MDPVPGLVKEEEEEGAHGRGDSPAVGAAPRPMEGLHDAGPPPFLTKTYDMVDDPNTDSIMSWSAGNNSFVVWDPHAFATVLLPRHFKHSNFSSFVRQLNTYGFRKVDPD
RWEFANEGFLRGQRHLLKNIRRRKPPAHTASNQQSLGSYLEVGHFGYDAEIDRLKRDKQLLMAEVVKLRQEQQNTKARLKAMEDRLRGTEQKQQQMTSFMARILRNPEF
LKQLIAKNGMRKELHDAISKKRRRRIDGGPEAYAVGASSSSLEQELPVVFDSHGSVELLAEGSVPVELLADGIPPDLEGSVELLADEIPPGLEGSVALLADGLEGSVEL
LVDGIPSDLDGSGIDSNGVTEPQDYGLGTCEAQQNRALGLFHDNFWEELLNKGLSVENDEPVNVDGMDVLSEKMGYLIPNSPTLST

TaHsfA2c
MDPVPSLVKEEEEEGAHGRGDSPGARAAPRPMEGLHDAGPPPFLTKTYDMVDDPNTDSIMSWSAGNNSFVVWDPHAFATVLLPRHFKHSNFSSFVRQLNTYGFRKVDPD
RWEFANEGFLRGQRHLLKNIRRRKPPAHTASNQQSLGSYLEVGHFGYDAEIDRLKRDKQLLMAEVVKLRQEQQNMKVHLKAMEDRLRGTEQKQQQMTSFMARILRNPEF
LKQLIAKNEMSKELHDAISKKRRRRIDGGPEAYAVGASSSNLEQESPVVFDSHGSVELLAEGSVPVPVELLADGIPPDLEGSVALLDDGIPPDLECSVELLVDGIPADL
NGSGIDANGVTEPQDFGLGTCEAQQNRVPGLFHDNFWEELLNKGLSGENDEPVNADGMDVLSEKMGYFIPNSPTLST

TaHsfA2d
GFRKVDPDRWEFANEGFLRGQRHLLRNIKRRKPTHGSQNQQSLASYLEVGNFGHDVEIDHLKRDKQLLMAEVVKLRQEQQNTRSGLQAMEKRLQGTEQKQQQMMSFLAR
VMQNPVFIRQLISQSEMRKELEDAISNKRRRRIDQGPEAVDSMGTGSTLEQGSHVMFEQQEPVDSFVNGVISDLESSSVDTKGAEVQQSVASSRSEQLRGRPSGELNDD
FWEDLLHEGGLGEEASNLVVPDDMNLLAEKLD

TaHsfA2e
GFRKVDPDRWEFANEGFLRGQRHLLKNIRRRKPPAHTASNQQSLGSYLEVGHFGYDAEIDRLKRDKQLLMAEVVKLRQEQQNTKARLKAMEDRLCGTEQKQQQMTSFMA
RILRNPEFLKQLIAKNGMRKELHDAISKKRRRRIDGGPEAYAMGASSSSLDQELPVVFDSHGSVELLAEGSVPVELLSDGIPPDLEGSVDLLGDEIPPGLEGSVALLAD
GLEGSVELLVDGIPSDLDAGIDSNGVTEPQDYGLGTCEAQQNRAPGLFHDNFWEELLNKGLSVENDEPVNVDGMDVLSEKMGYLIPNSPTLST

TaHsfA2f
GFRKVDPDRWEFANEGFLRGQRHLLRNIKRRKPTHGSQNQQSLGSYLEVGNFGHDVEIDHLKRDKQLLMAEVVKLRQEQQNTRSDLQAMEKRLQGTEQKQQQMMSFLAR
VMQNPMFIRQLISQSEMRKELEDAISNKRRRRIDQGPEAVDGMGTGSTLEQGSQVMFEKQEPVDSLVNGVISDLESSSVDTKGAEVQQSVASSRSEQLRGRPSGELNDD
FWEDLLHEGGLGEEASNLVVPD
DMNLLAQKMD

TaHsfA2g
MSHRMMMNPVKVEGDGRAGGGGAPRPMDGLGDAGPTPFLAKTYDMVDDPATDAVVSWSATNNSFVVWDPHLFATVLLPRYFKHGNFSSFVRQLNTYGFRKVDPDRWEFA
NEGILRGQXHLXRNIKRRKPNMGS

TaHsfA2h
MDPVPSLVKEEEEEEGAHGRGDSPGARAAPRPMEGLHDAGPPPFLTKTYDMVDDPNTDSIMSWSSGNNSFVVWDPHAFATVLLPRHFKHSNFSSFVRQLNTYGFRKVDP
DRWEFANEGFLRGQRHLLKNIRRRKPPAHTASNQQSLGSYLEVGHFGYDAEIDRLKRDKQLLMAEVVKLRQEQQNMKVHLKAMEDRLRGTEQKQQQMTSFMARILRNPE
FLKQLIAKNEMSKELHDAISKKRRRRIDGGPEAYAVGASSSNLEQESPVVFDSHGSVELLAEGSVPVPVELLADGIPPDLEGSVALLDDGIPPDLECSVELLVDGIPAD
LNGSGIDANGVTEPQDFGLGTCEAQQNRVPGLFHDNFWEGLLNKGLSGENDEPVNADGMDVLSEKMGYFIPNSPTLST

TaHsfA2i
MDPFHGIVKEEEFDFAGAAADGYSPSSWGSSPSSWGSSQSSWAGGGALAELPRPMDGLGEAGPTPFLNKTYEVVDDHSTDTIVSWGVAGNSFVVWDAHAFSMVLLPRYF
KHCNFSSFVRQLNTY

TaHsfA3a
MDHTHTGINTTPAAVTASASMDAALLLEPKLEMMQQQQPSPAGHYAALDHHLIPPPPALVVPCEPPRPLEALLQGPQLPPFLSKTYDLVSEPQLDGVISWGPAGNSFVV
WNPSTFARDVLPHNFKHNNFSSFVRQLNTYGFRKVHADRWEFAHEGFLRGSKHLLKTIVRRRSSPTQQSSLQPGSSIFRKIQSGSSGESTVDPELRSLRREKNALLQEV
ARLKEEHNQTIEHMNALNQRLETAEDRQKQVVSFLAKLLQNPDFLRQLKMHRERRDGIDSARVKRKFLKHVPHGSRDSGDSSSPRTAESTCSPAHPVAHDAIADLQSFL
LEDTDLSDGMMPGNFGLDGVEASEDIGALVQVFDQQDPGTGAELLGIPPVSGAAHCQDLTVGRSKGKNVMCPGGTDGTSSQADCLVPLPGNVGKLMDADGEQIWGADTF
FQSSCSGTSQQTYVSDPYLIAIPDKHEKFWEVDFEALDDGDLHLDKCVIGDPALEQHRGNMKP

TaHsfA3b
YRDVELLEILQFLLLLISQDSTFGRSKGKNVMCSGGTDGTSSQANCLVSLPGNVCKLMDADGEQIWGADTFFQSSCSGTSQQAYVSDPYLMEIPDKPERFWELDSRFGG
TG

TaHsfA4a

MEGSGGSSSLPPFLTKTYEMVDEPATDAVVAWTPSGTSFVVLSQADFCRDLLPKYFKHNNFSSFVRQLNTYGFRKVDPEQWEFANEEFIRDQRHRLKNIHRRKPIFSHS
SHTQGAGPLADSERRDYEEEIERLKCDNASLKLQLERKKTDMESKMKVLEDKLFAIEGQQKNLISYVREIVNAPGFISSLIEQSDHHGKKRRLPKPISFHEDASTQGNQ
IMHCDMVNSPTHELFRASFDKMESSLNSLENFFKEASEAFGNDVSYDGDVPGHSSAVVLTELHSSGESEPHAQSPPSMMHTCSAGVGDSHSSRDIAESASCPESSPLPE
AHSRADSRSKVSEIDVNLEPAVTETGPSRDQQPTQDPPADANDGFWQQFLTEQPGLSHAHQEAQSERRDREANQTTAGDRGSFWWGKSVEQMTEKLGHLTSAEKT

TaHsfA4b
MEGSGGSASLPPFLTKTYEMVDEPATDAVVAWTPSGTSFVVLSQADFCRDLLPKYFKHNNFSSFVRQLNTYGFRKVDPEQWEFANEEFIRDQRHRLKNIHRRKPIFSHS
SHTQGAGPLADSERRDYDEEIERLKCDNASLKLQLERKKTDMESKMKALEDKLFAIEDQQKNLISYVREIVNAPGFFSSFVEQSDHQGKKRRLPKPISFHEDTSTQGNQ
IMHCDLVNSPTHELFRASFDKMESSLNSLENFFKEASEAFGNDVSYDGDVPGHSSAVVLTELHSSAESEPLHSSAESEPHEQSPPSMMHTCSAGVGDSHSSRDIAESAS
CPESPPLPEAHSRADSRAKVSEIDVNLEPAVTETGPSRDQQPTQDPPADANDGFWQQFLTEQPGLSHAHQEAQSERRDREANQTTAGDRGSFWWGKSVEQMTEKLGHLT
SAEKT

TaHsfA4c
MDMDMDMEGGSQSQPQGASLSPAPFLIKTYEMVEDPATSRVVSWGPGGASFVVWNPPDFSRDLLPKYFKHNNFSSFIRQLNTYGFRKIDPERWEFANDDFIRGHMHLLK
NIHRRKPVHSHSPQNQVNGPLAESERREYEEEISRLKHENSVLVAELQRQAQQQCGLSWLMQSLEERLLAMEQRQADVVASVRDIQQRRRGDVHPGQQTMLELEPTDHF
SKKRRVPTIGFFAEEPAAAAEEHLRATGGETPGMVMVNAEPFEKMELALVSMEKLVQRAGNYAPSEDMFSAATASASAAPSTDDPAHAGLQEAPVETGVDLQPTSPELA
EPPGYAVQSPMLLFPEIQEDKRNTTAEVDMSSEASTTDTSQDETNAEAETPHEQAVANDLFWERFLTDTPKPLAVEDSHESKDGVKMGLDCYWFGHRNNVDQITQQMGH
LASAQYAHVCRQ

TaHsfA4d
MDIDMEGGSQPQPQGASLSPAPFLIKTYEMVEDPATNRVVSWGPGGASFVVWNPPDFSRDLLPKYFKHNNFSSFIRQLNTYGFRKIDPERWEFANDDFIRGHMHLLKNI
HRRKPVHSHSPQTQVNGPLAEAERREYEEDISRLKHENSVLVAELQRQARQQCGLSWLMQSLEDRLMAMERRQADVVASVRDTLQRRRGGVHPGQQTMLELEPTDHFSK
KRRVPRLGFFVEEPAAAAAEEQRVPHLRAMGGETPGMVMVNAEPFEKMELALVSMEKLVQRAGDYASSEDMYNAAAAAPSTDDPAHADLQAAPVNLQPSSPEIAESPGY
AVQSPMLLFPEIHEDKHKTMAEVDMSSEASTTDTSQDETTAEAEAETGVPHEPAVANDLFWERFLTDTPKPLAVEDRNESKDDVKTGLDCYWFGHRNNVEQITEQMGHL
ASAQKT

TaHsfA4e
MEMDMEGGSQPQPQGASLSPAPFLIKTYEMVEDPATSRVVSWGPGGASFVVWNPPDFSRDLLPKYFKHNNFSSFIRQLNTYGFRKIDPELWEFANDDFIRGHMHLLKNI
HRRKPVHSHSPQNQVNGPLAESERREYEEEISRLKHENGLLVAELQRQAQQQCGLSWLMQSLEDRLTAMEQRQANVVASVRDTLQRRRGGVHPRQETMMLELEPTDHFS
KKRRVPTIGFFVEEPAEEQRVPHLRAMGGETPGMVMVNAEPFEKMELALVSMEKLVRRAGDYASSSEDMYNAAAAAAPSTDDPAHADLQAAPVETGANLQPSSPELAES
PGYAVQSPMLLFPEIHEDKRNTTAEVDMSSEASTTDASQDETTAEAEAETGVPHEPAVANDLFWERFLTDTPKPLAVEESHGPEDDVKTGLDCYWFGHRNNVDQITEQM
GHLASAQKT

TaHsfA4f
PQVLKHNNFSSFVRQLNTYGFRKVDPEQWEFANEEFLRDQRHRLKNIHRRKPIFSHSSHTQGAGPLADSERRDYEEEIERLKCDNASLNLQLERKKTDMESKMKALEDK
LFAIEDQQKNLISYVREIVNAPGFLSSFVEQSDHHGKKRRLPKPISFHDDACTQGNQIMHCDLVNSPAHELFRSSFDKMESSLNSLENFFKEASEAFGNDVSSDGDVPG
HSSAVVLTELHSTGESDPHAQSPPSMMHTCSAGVGDSHSSRDIAESASCPESPLLPQAHSRADSRAKVSEIDVNLEPAVTENGPSRDQQPTQDLPADANDGFWQQFLTE
QPGLTHAHQEAQSERRDREANQTTAGDRGSFWLDKSSIEQMTKKLGHLTSAEKT

TaHsfA5a
MEGSGAVGARGGGGGGPAPFLLKTYEMVDDPATDAVVSWSDASDTSFVVWNSPEFAARLLPAYFKHSNFSSFIRQLNTYGFRKIDPERWEFGNEYFVKGQKHLLKNIYR
RKPIHSHSHQPGALPDNERSFFEDEIDRLAREKANLQAELWKSKQQESGTMFQIEALERRAVDMEQRQGKMIAFLQQASKNPHFVSKLVKMAEASSMFADALHKKRRLS
GLDYAVEATETASFCDDHSATSRQEMGNLLNQHFSDKLKLGLCSAAAESNLVTLSTQSSHEDNGSPHGRHPPVHDGMGTGCLPLVPQIMELSDTGTSICPSKSSFFAPA
VNDEGLSPCHLSLTLASCSMDVDRGQASNADGSTTINEGSDNPPEATATAMGGNPKASVDAGTAEAPTPREDTWVAAEPAAPVKANDNFWEQFLTERPGDASSGPRDPS
MDKEQTEGGGKDDREVTEQLKL

TaHsfA5b
MEGSGTAGARGGGGGGPAPFLLKTYEMVDDPSTDAVVSWSDASDTSFVVWNSPEFAARLLPAYFKHSNFSSFIRQLNTYGFRKIDPERWEFGNEYFVKGQKHLLKNIYR
RKPIHSHSHQPGALPDNERSFFEDEIDRLAREKANLQAELWKSKQQESGTMFQIEALERRAVDMEQRQGKMIAFLQQASKNPHFVGKLVKMAEASSMFADALHKKRRLS
GLDYAVEATETASFCDDHSPTSRQETGNLLNQHFSDKLKLGLCSAAAESNLVTLSTQSSHEDNCSPHGRHPPGHEGMGTGRLPLVPQMMELSDTGTSICPSKSSFFTPA
VNDEGLSPCHLSLTLASCSMDVDRGQASNADGSTTVDEGSDNPPEATTAAMGVNQKASVDAGTAEALRPREDMRVATEPAAPVKANDNFWEQFLTERPGDASSGPRDPS
MDKEQTERGEKDDTEQLKL

TaHsfA6a
MDRVLLPVRVKEEWPPPPPEEEEELEHGGLAPRPMEGLHETGPPPFLTKTFDLVADPATDGVVSWGRAGNSFVVWDPHLFAAVLLPRFFKHSNFSSFVRQLNTYGFRKI
DPDRWEFANEGFIRGQRQLLKMIKRRRPWSYLPSPQHQALGSCLEVGQFGLDDEIEVLKRDKNALLEEVVKLRQEQQSSRADMRAMEERLHRVEQKQLQMMGFLARAIQ
NPDFFLQLIQQQDKLKDLEDAYSNKRRRSIDVMPFLGPEGASQSEPLESTFIFEDREFSELENLAMNIQGIRKGMEGDRGGRDQGCGEAELTDDFWEELLSEGMRDEAE
MVELERRRSRYVDA

TaHsfA6b
GFRKIDPDRWEFANEGFIRGQRQLLKMIKRRRPMSYLPSSQQQALGSCLEVGQFGFDEEIEVLKRDKNALLAEVVKLRQEQQSSRADMRAMEERLHHVEQKQLQMMGFL
ARAIQNPDFFLQLIQQQNKLKDLEDGYPTKRRRPIDVMPFLGPEGTSQSEPLESTFIFEDREFSELENLAMNIQGIRKGMEDDRGGRNQGCGEAELTDDFWEELLSEGM
RDEAEMLELERRRSRCFDA

TaHsfA6c
GFRKVDPDRWEFANEGFLRGQRHLLKTIKRRKPPSNAPSSQQQALTSCLEVGEFGFEEEIDRLKRDKNLLITEVVKLRQEQQATKDNVQAMEGRLRAAEQRQAQMMGFL
ARAMRNPHFFQQLVQKQDKRKELEDAISKKRRRPIDNAPFYGSGATTSQSEQLDSQFLFDSGVLSEPGMNGMENLAQNIQELGQGKTDEEKKDEANGQLDINSDFWAEL
FSDDFGDEDGSGLSELEGRRPEDIDELGQQLGYLSSTSPQ

TaHsfA6d
GFRKVDPDRWEFANEGFLRGQRHLLKMIKRRKPPSNAPSSQQQALTSCLEVGEFGFEEEIDRLKRDKNLLITEVVKLRQEQQATKDNVQAMEGRLRAAEQRQAQMMGFL
ARAMCNPHFFQQLVQKQDKRKELEDAISKKRRRPIDNAPFYGPAATTSQSEQLDSQFLFDSGVLSEPGMNGMENLAQNIQELGQGKTDEEKKDEANGQLDINNDFWAEL
FSDDFGDGDGSGLSELEGRRPEDIDELAQQLGYLSSTSPQ

TaHsfA6e
MDAMPPEGIVKEEEVLLHEEHTHPQAGPQQQQQRQDGALPRPMEGLHEAGPPPFLTKTYDLVEDPATDQVVSWGRAGNTFVVWDPHVFAEALLPRLFKHSNFSSFVRQL
NTYGFRKVDPDRWEFANEGFLRGQRHLLKMIKRRKPPSNAPSSQQQALTSCLEVGEFGFEEEIDRLKRDKNLLITEVVKLRQEQQATKDNVQAMEGRLRAAEQRQAQMM
GFLARAMRNPHFFQQLVQKQDKRKELEDAISKKRRRPIDNAPFYGPGATTSQSEQLDSQFLFDSGVLTEPGMNGMENLAQNIQELGQGKTDEEKKDEANGQLDINNDFW
AELFSDDFGDGDGSGLSELEGRRPEDIDELAQQLGYLSSTSPQ

TaHsfA6f
MDRVLLPVRVKEEWPPPLPEEEEELEHGGLAPRPMEGLHETGPPPFLTKTFDLVADPATDGVVSWGRAGNSFVVWDPHLFAAVLLPRFFKHSNFSSFVRQLNTY

TaHsfA7a
MDYAAAVTMDIKQEEPEMVVLDDDDDGDAGCCLAPTPLDLAAAAAVAPFLAKTFDMVEDPATDAVVSWGAARNSFVVWDPHAFAARLLPRHFKHANFSSFLRQLNTYGF
RKVNPDRWEFANTGFLGGQRHLLAGIRRRRGADTGRRPAAALSPSSCAESAGGFGPVEGELEQLRRDREALKRELAGLKRQQEEARATLLDMERRVQGTERRQEQCKAF
LARAVRNPAFLANLARRNGLTAAAPAPAVDGKKKRRLLDVIPSPPPAEDGFTFEELALAAGVVEEAAAPTQGAGAGGVTTDMIWYELLEEGQAEIDVDVEDLVAAAGDM
EPWGFGAEEVQDLMQQIDSFACSPSC

TaHsfA7b
MDSAAAVTMDVKQEEPEMVVLDDDDGDAGCCLAPTPLDLSAAAAVPPFLAKTFDMVEDPATDAVVSWGAARNSFVVWDPHAFAARLLPLHFKHANFSSFLRQLNTYVSG
FRKVNPDRWEFANTGFLGGQRHLLAGIRRRRGADTGRRPAAALSPSSSCAEGAGGFGPVEGELERLRRDREALKRELAGLKRQQEEARATLLDMERRVEGTERRQEQCK
AFLARAVRSPAFLANLARRNGLSAAAPASVIDGKKKRRLLNANSSPPPAEDGFTFEELALAAGVVEEAAAPTRSGVTTDMIWYELLEEGQAEIDVEVEELVAAAGDMEP
WEFGEEEVQDLMQQIDSFAGPPSC

TaHsfA8a
MGSSKGSPPPSAAGPSSAGNAPAPVGPAPRPPEVAPFLTKVYDMVSDAATDKVMSWTDAGNSFVIWDAHAFERDLLSRHFKHRNFSSFIRQLNTYGFRKVDPDRWEWAN
EGFLRGQKHLLKIIKRKKRPQEAGRELEKAPVKAAPGTENIEIGRYGGLVKEVETLKRDKSLLMQQLVDLRHYQQSSNLEVQSLIQRLQVMEQNQKQMMALLSIVVQNP
SLLNQLVQQQQQQQRRTNWYEDGNKKRRFPALEQGPVTDYETSGAGTEIIQYRPPAPETSSQVIPDEAFLSATTQPISSPALNMPMDIDTQTTSDNLNTQGSSGDIFAD
MPALPDFDDMHLWFGEDGEPTLTIQDYDESPQSEQDCQMEAQHNYNNPQHADVITEA

TaHsfA8b
GFRKVDPDRWEWANEGFLRGQKHLLKIIKRKKRPQEAGRELEKAPVKAAPGTENIEIGRYGGLVKEVETLKRDKALLMKQLVDLRLYQQSSNLEVQSLIQRLQVMEQNQ
KQMMALLAIVVQNPSLLNQLVQQQQQQRRNNCVYEDGNKKRRFPALEQGPVTDHETSGAGAEIIQYRPPVPETSSQVIADEAFLSATAQPISSPALNMPMDIDTQTTSD
NLNTQGSSGDIFADMPALPDFEDMHLWFNEDGEPTLTIQDYDESPQSGQDCQMEAQHNYNYPQHADAITEA

TaHsfA8c
GFRKVDPDRWEWANEGFLRDQKHLLKIIKRKKRPQEAGRELEKAPVKAAPGTENIEIGRYGGLVKEVETLKRDKALLMKQLVDLRLYQQSSNLEVQSLIQRLQVMEQNQ
KQMMALLAIVVQNPSLLNQLVQQQQQQRRNNCVYEDGNKKRRFPALEQGPVTDHETSGSGAEIIQYRPPVPETSSQVIADEAFLSATAQPISSAALNMPMDIDTQTTLG
NLNTQGSSGDIFADMPALPDFEDMHLWFSEDGEPTLTIQDYDEFPQSGQDCQMEAQHNYNNPQHADAITEA

TaHsfB1a
MAGAAAQQQQQKGGGAVRVGGGGPAPFLTKTHQMVEERGTDEVISWGEQGRSFVVWKPVELARDLLPLHFKHCNFSSFVRQLNTYGFRKVVPDRWEFANENFRRGEQGL
LSGIRRRKATATTTTPQSSKTSGTGVNVAFPPPLPALRPASASTSGTGNDHSSSSASSPTRPDLSSENEQLRKDNHALAAELALARRHCEELLGFLSRFLDVRQLDLRL
LMDGDMQGAAGAGGARSADQEHCCEKKVKLFGVILKDASARKRGRCDEAAASERSMKMTRIGEPWVGVPSSCPARCGGGN

TaHsfB1b
MAGAAAQQQQKGGDGGGVVRIGGGGPAPFLTKTHQMVEERGTDEVISWSEHGRSFVVWKPVELARDLLPLHFKHCNFSSFVRQLNTYGFRKVVPDRWEFANENFRRGE
QSLLSGIRRRKATATTIPQSSKTCGTGVNVAFPRPLPVLPPASVSTSGTGNDHSTSSASSPTRPDLSSENEQLRKDNHALAAELALARRHCEELLGFLSRFLDVRQLDL
RLLMDGDMQGAAGGARSADQEHCCEKKVKLFGVILKDASARKRGRCDEAAASERSMKMTRIGEPWVGVPSSCPARCGGGN

TaHsfB1c
MAGAAAQQQKGGSGGGGGAVRVGGGGPAPFLTKTHQMVEERGTDEVISWSEHGRSFVVWKPVELARDLLPLHFKHCNFSSFVRQLNTYGFRKVVPDRWEFANENFRRGE
QSLLSGIRRRKATGTTTPQSSKTCGTGVNVAFPPPLPALPPASASTSGTGNDHSTSSASSPTRPDLSSENEQLRKDNHALAAELALARRHCEELLGFLSRFLDVRQLDL
RLLMDEDMQGAAGGARSADQEHCCEKKVKLFGVVLKDASARKRGRCDEAAASERSMKMTRIGEPWVGVPSSCPARCGGGN

TaHsfB2a
MGEQAAAVSSGETPATAEVGTGLGQRSLPTPFLNKTYQLVDDPAVDDVISWSEDGSAFIVWRPAEFARDLLPKYFKHNNFSSFVRQLNTYGFRKIVPDRWEFANDCFRR
GEKRLLCDIHRRKVTPTVAATAAVTVAAAAAIPVALPVAKRQGSPVLSGDEQVLSSSSSPEPPFLNQYAPSYSGSGGVASGDLGEENERLRRENSRLTRELGQMKKLCN
NIFVLMSKYTDGQQTDAANADLRRRRRRELLWVGRDHGAPTSAGAPKLWPSCQIAPHGCRFGRDEEEKSRASRRLHRAKRCPTPQEPTSKNARSEA

TaHsfB2b
MGEQAAAVSSGETPATAEVGTGLGQRSLPTPFLNKTYQLVDDPAVDDVISWSEDGSAFIVWRPAEFARDLLPKYFKHNNFSSFVRQLNTYGFRKIVPDRWEFANDCFRR
GEKRLLCDIHRRKVTPTVAATAAVTVAAAAAIPVALPVAKRQGSPVLSGDEQVLSSSSSPEPPFLNQYAPSYSGSGGVASGDLGEENERLRRENSRLTRELGQMKKLCN
NIFVLMSKYTDGQQTDAANATSAAADVGNCSGESAETTALPPPPVLELLPSCQNAPTAADLGAEDEEEKMSARLFGVCIGRKRMRHDGEDRTRRGAAAEVKPEPMDAQQ
PSGMDGHTPDVQAWPIYRPRPVYQPLRASDGSNCYSGSDDHNGSNSR

TaHsfB2c
MSAEHGAAAADGAGAEPPLPAPMPGLATSADAAGQRSLPTPFLTKTYQLVEDPAVDDVISWGEDGSTFVVWRPAEFARDLLPKYFKHNNFSSFVRQLNTYGFRKIVPDR
WEFANDCFRRGEKRLLCDIHRRKVVQSAGLAAAAAAAAAGAVTVATAAIPMALPVTRSGSPEPQLSSEEQVLSSNSGSAEELPLAAPSGSGSGLGGAAAGSSSGDMGEE
NDRLRRDNARLTRELGQMKKLCNNIVSLMSKFASSQQPDGGPGSLSSVVNCSGESALAPPPPLPAGILDLMPSCSALATAAGLAVDGGPETDARLFGVSIGLKRARDEE
EDGDGEELPNGDGAGVKPEEAAERRADGGSEDRQSWPIYRPKPVYRACNGQDGAGAGAGSGSDQDRSNSR

TaHsfB2d
MSAEHGAAAADGAGAEPPPPAPMPGLVASADAAGQRSLPTPFLTKTYQLVEDPAVDDVISWGEDGSTFVVWRPAEFARDLLPKYFKHNNFSSFVRQLNTYGFRKIVPDR
WEFANDCFRRGEKRLLCDIHRRKVVQSAGLAAAAAAAAAGAVTVATAAIPMALPVTRSGSPEPQLSSEEQVLSSNSGSAEELPLAAPSGSGSGLGGAAAGSSSGDMGEE
NDRLRRDNARLTRELGQMKKLCNNIVSLMSKFASSQQPDGGPGSLSSVVNCSGESALAPPPPLPAGILDLMPSCSALATAAGLAVDGGPDTDARLFGVSIGLKRARDEE
EDGDGGELPNSNGDGADVKPEEAAERRPEGGGSEERQSWPIYRPKPVYRACNGQDGAGSGSDQDRSNSR

TaHsfB2e
MAAEHGAAAAAADRAGAEPPPPAPMPGLVASVDAAGQRSLPTPFLTKTYQLVEDPAVDDVISWGEDGSTFVVWRPAEFARDLLPKYFKHNNFSSFVRQLNTYGFRKIVP
DRWEFANDCFRRGEKRLLCDIHRRKVVQSAGLAAAAAAAAAGAVTVATAAIPMALPVTRSGSPEPQLSSEEQVLSSNSGSAEELPPAAPSGSGSGLGGAAAGSSSGDVG
RGERRLRRDNARLTRELGQMKKLCNNIVSLMSKFASSQQPDGGPGSLSSVVNCSGESALAPPPPLPAGILDLMPSCSALATAAGLAVDGGPETDARLFGVSIGLKRARD
EEEDGDGEELPNSNGDGADVKPEEAANRRPDGGSEDRQSWPIYRPKPVYRACNGQAGAGAGAGSDQDRSNSR

TaHsfB4a
MAFLVERCGEMVVSMEMGSGAAGAHGAGGGVAGKPVPAPFLTKTYQLVDDPCTDHIVSWGEDDATFVVWRPPEFARDLLPNYFKHNNFSSFVRQLNTYGFRKIVADRWE
FANEFFRKGAKHLLAEIHRRKSSQPPLPSMVPHQAAYHHHYHLGNTLSPPPPPPPAAHHHHHPVYQHFQEEPAAAATSHGGGNGGGDFLAALSEDNRQLRRRNSLLLSE
LAHMKKLYNDIIYFLQNHVAPVTSPSSVASQRHMPLPGTAAVATSNSCRLLELDMDVDRDSPAAEEDDTVKLFGVALHHGKKKRAHSEERGDVVAHDLGSEV

TaHsfB4b
MAFLVERCGEMVVSMEMGSGAPGAHGGGGIAGKPVPAPFLTKTYQLVDDPCTDHIVSWGEDDATFVVWRPPEFARDLLPNYFKHNNFSSFVRQLNTYGFRKIVADRWEF
ANEFFRKGAKHLLAEIHRRKSSQPPPPSMVPHQAAYHHHYHLGNTFSPPPPPPPAAHHHHHHPVYQHFHEEPAAAAASHGGGNGGGDFLAALSEDNRQLRRHNSLLLSE
LAH

TaHsfB4c
MERCGSWSESDAAAQAAAQKAVPAPFLTKTYQLVDDPATDHIVSWGDDRVSTFVVWRPPEFARDILPNYFKHNNFSSFVRQLNTYGFRKVVPERWEFANEFFRKGEKQL
LCEIHRRKTSGSTTSSSPPPFFAPPHFPLFHPGVSVAHRHQQFMGEDGAMAAHAGMGGPFMQPHWREQGAPRLLALGGPAAPSPGVEGNGRASSAAVL

TaHsfC1a
MDGLHTELALGLIGCGHGDLQTAPFVAKTYQMVCDPRTDALVRWGKGNNSFLVTDVAGFSQLLLPCFFKHGNFSSFVRQLNTYGFRKVHPDRWEFAHESFLRGQTHLLP
RIVRRKKRGEAGAGASCSSAVGGGEQHQHVVANMGDQVEEEEDEEGREALLEEVQRLRQEQTAIGEQLAKMSRRLQATERRPDRLMSFLSKLAEDPNATSLHLLEQAAE
KKRQRMQCPSRDFTSFPVALPLHPAPSPPPPPLLALGDPTMGGVRVWQWAEPMPLALTTFEQPSASSGVQQVPEFEGGRSGSSMGITDGGTAVETPFPFCLLGQCFF

TaHsfC1b
MGSECKGHQPQDDGGVAPFVAKTFHMVSDPATDAVVCWGGASNTFLVLDPAAFSDFLLPSYFKHRNFASFVRQLNTYGFRKVDPDVWEFAHESFLRGQAKLLPLIVRKK
KRAGRELCEEEEEVRGTIQAVQRLRDERRGMEEELQDMDRRLCAAENRPGQMMAFLGKLADDPGVVLRAMVAKKEELAAAGAGGKDSSPDKRRRIGADAGRGADAADQA
AQSRAVPFPFSNLGQVFY

TaHsfC1c
MGSECKGHQPQDDGGVAPFVAKTFHMVSDPATDAVVCWGGASNTFLVLDPAAFSDFLLPSYFKHRNFGSFVRQLNTYGFRKVDPDVWEFAHESFLRGQEKLLPLIVRKK
KRAGAGAAGRELCVEEEEVRGTIQAVQRLRDERRGMEEELQAMDRRLCAAENRPGQMMAFLGKLADDPGVVLRAMVAKKEELAAAGAGGKDSSPDKRRRIGADADQAAQ
SRAVPFPFCNLGQVFY

TaHsfC1d
MGSECKGHQPQDDGGVAPFVAKTFHMVSDPATDAVVCWGGASNTFLVLDPAAFSDFLLPSYFKHRNFASFVRQLNTYGFRKVDPDVWEFAHESFLRGQAKLLPLIVRKK
KRAGAGAAGRELCEEEEEVRGTIQAVQRLRDERRGMEEELQAMDRRLRAAENRPGQMMAFLGKLADDPGVVLRAMVAKKEELAAAGAGGKYSSPDKRRRIGADAGRGAD
AADQAAQSRAVPFPLSNLGQVFY

TaHsfC1e
MDGLHTELALGLIGCGHGDLQTAPFVAKTYQMVCDPRTDALVRWGKGNNSFLVTDVAGFSQLLLPCFFKHGNFSSFVRQLNTYGFRKVHPDRWEFAHESFLRGQTHLLP
RIVRRKKRGEAGVGASCSSAVGGGEQQQHVVANMGDQGEEEEDEEGREALLDEVQRLRQEQTAIGEQLAQMSRRLQATERRPDRLMSFLSRLAEDPNATSLHLLEQAAE
KKRQRMQCPSRDFTSFPIALPLQPAPSPPPPPLLALGDAAMGGVRVWQWAEPMPLKLTTVEQPSGSSGVQQVPEFEGGRSGSGMGITDGGTAVETPFPFCLLGQCFF

TaHsfC2a
MAAASVGGGAAPFVWKTYRMVEDPGTDGVIGWGKGNNSFVVADPFVFSQTMLPAHFKHNNFSSFVRQLNTYGFRKVDPDRWEFAHGSFLRGQTHLLRNIVRRGTPAPGG
GGKRKDAGAAGLTDDDMTMVATEVVRLKKEQSTIDDRVAAMWRRVQETERKPKQMLAFLLTIVGDRDTLQRLVGNSGNAAGGDQEPVEGGEKRARLLLDGDFGNVSAFG
PDAVDFAGFYTDDAFANAPVPVEAAAGSGGGGAGCTFAFGVDSGY

TaHsfC2b
MAAAAASVGGGAAPFVWKTYRMVEDPGTDGVIGWGKGNNSFVVADPFVFSQTMLPAHFKHNNFSSFVRQLNTYGFRKVDPDRWEFAHGSFLRGQTHLLRNIVRRGTAVG
GGGGGKRKDAVAAGLTDDDMTMVATEVVRLKKEQNTIDDRVAAMWRRVQETERKPKQMLAFLLTVVGDRDTLQRLVGNSGNAAGGEQGPVEGGEKRARMLLDGDFGNVS
AFGPDAVDFAGFYTDDAFASVPVPVEAAAGPGGGGAGCTFAFGVDSGY

TaHsfC2c
PRVGGRESSSSSGSSSGASAAAGVAPFVAKTYGMVDDRATDAGVAWGSAGNSFVVADPFAFSEMLLPAHFKHSNFSSFVRQLNTYGFRKVDPDRWEFAHASFLRGQTHL
LPRIVRRQSSGTGGRRGKDDREDEDGSSAMLAMEVVRLRREQRATEERVAAMWRRVQETERRPKQMLGFLLKVVGDPDVVRRLAGSGQEEVARVNRPRLLLDSREEQRK
PVDGQSYHNHSNLNLVEACVLEPSVELYYAGGDAQADGGGGGHPPYAFHVNSGY

TaHsfC2d
MSGAGGMECSGSGGTVAPFVAKTFAMVDDPATDAVVRWGPASNSFVVADPFTFSHALLPAHFKHANFSSFVRQLNTYGFRKVDPDRWEFAHASFLRGQTHLLPRIVRRR
SGGKGGKEDADGEEDEDISSKMLAMEVVRLKEEQRATEDRLASMRRRVQDAERRPKLMLAFLLKVVGDPDVLRRLVGNSSCGGGLFPGEGAEAKRPRLLLDGEVQVGKK
KMRVDGDGLSCGINRQEAFVREPRVDFTGFYTGGDGFSDVPVANDLPYAFPVDTGY

TaHsfC2e
MSSGGGIGCSGSGDGDGGMVAPFVAKTYAMVDDPATDAVVAWGPASNSFVVADPFAFSRALLPAHFKHANFSSFVRQLNTYGFRKVDPDRWEFAHACFLRGQTHLLPRI
VRRRSGGKRGKEDADEEDEDISSKMLAMEVVRLKEEQRATEDRLAAMWRRVQDAERRPKLMLAFLLKVVGDPDVLRRLVGNSSSGGVLFPGEGAEAKRRRLLLDGEGQV
SKKKMRVDGDGLLCGINRQDAFVREPRVDFTGFYTGGDGFSEVPVDDPPYAFPMDGGY

TaHsfC2f
MSRDGGGMVAPFVAKTYAMVDDPATGAVVAWGPASNSFVVADPFAFSEMLLPAHFKHSNFSSFVRQLNTSGFRKVDPDRWEFAHASFLRGQTHLLPRIVRRSGGGSRRA
KEDRDEEQGISSTALAMEVVRLRNEQRATEESMAAMWRRVQDAERRPKLMLAFLLKVVGDPDALHRLIGNSSSDGGLFPGEGAEAQRPRLLLNREVQVGEQMHIDGEGQ
LYGISRQDAFIQESPADFTGFYTGGDGFSDVPTDDPPYAFTVDGSY

TaHsfC2g
MSTGGAGKGCSGSDGGMVAPFVAKTYAMVDDPATDAVVAWGPASNSFVVADPFAFSRALLPAHFKHANFSSFVRQLNTYGFRKVDPDRWEFAHACFLRGQTHLLPRIVR
RRTGGGRRAKDSHDEEEDEDISSKMLAMEVARLKEEQRATKDRLAAMWRRVQDAERRPKLMLAFLLKVVGDPDVLRRLVGNSGGGGGLFPGEGAEAKRPRLLLDGEAQV
GKKMRIDGDGMLCGINRQEAFVREPRVDFTGFYTGGDGFSEVPVDDPPYAFRVDTGY

Supplementary Figure S1. The deduced protein sequences of TaHsfs.

```
TaHsfA1a   PpPFLmKTYDMVDDPATDAVVSWGpAnN-SFiVWntpEFARdLLPKYFKHNNFSSFVRQLNTYGFRKVDPDkWEFANEGFLRGQKHLLKtInRRK
TaHsfA1b   PpPFLmKTYDMVDDPATDAVVSWGpAnN-SFiVWntpEFARdLLPKYFKHNNFSSFVRQLNTYGFRKVDPDkWEFANEGFLRGQKHLLKtInRRK
TaHsfA1c   PpPFLmKTYDMVDDPATDAVVSWGpAnN-SFiVWntpEFARdLLPKYFKHNNFSSFVRQLNTYGFRKVDPDkWEFANEGFLRGQKHLLKtInRRK
TaHsfA2a   PtPFLaKTYDMVDDPATDAVVSWtatsN-SFVVWDPhlFAtlLLPsYFKHsNFSSFVRQLNTYGFRKVDPDRWEFANEGFLRGQRHLLRnIkRRK
TaHsfA2b   PpPFLTKTYDMVDDPnTDSimSWsagnN-SFVVWDPhaFAtvLLPRhFKHsNFSSFVRQLNTYGFRKVDPDRWEFANEGFLRGQRHLLKnIrRRK
TaHsfA2c   PpPFLTKTYDMVDDPnTDSimSWsagnN-SFVVWDPhaFAtvLLPRhFKHsNFSSFVRQLNTYGFRKVDPDRWEFANEGFLRGQRHLLKnIrRRK
TaHsfA2d*                                                                 GFRKVDPDRWEFANEGFLRGQRHLLRnIkRRK
TaHsfA2e*                                                                 GFRKVDPDRWEFANEGFLRGQRHLLKnIrRRK
TaHsfA2f*                                                                 GFRKVDPDRWEFANEGFLRGQRHLLRnIkRRK
TaHsfA2g   PtPFLaKTYDMVDDPATDAVVSWsatnN-SFVVWDPhlFAtvLLPRYFKHgNFSSFVRQLNTYGFRKVDPDRWEFANEGiLRGQxHLxRnIkRRK
TaHsfA2h   PpPFLTKTYDMVDDPnTDSimSWssgnN-SFVVWDPhaFAtvLLPRhFKHsNFSSFVRQLNTYGFRKVDPDRWEFANEGFLRGQRHLLKnIrRRK
TaHsfA2i*  PtPFLnKTYEvVDDhsTDtiVSWGvAGN-SFVVWDahaFsmvLLPRYFKHcNFSSFVRQLNTY
TaHsfA3a   lpPFLSKTYDlVsePqlDgViSWGpAGN-SFVVWnPstFARdvLPhnFKHNNFSSFVRQLNTYGFRKVhaDRWEFAhEGFLRGsKHLLKtIvRRr
TaHsfA4a   lpPFLTKTYEMVDePATDAVVaWtpSGt-SFVVlsqaDFcRdLLPKYFKHNNFSSFVRQLNTYGFRKVDPeqWEFANEeFIRdQRHrLKnIhRRK
TaHsfA4b   lpPFLTKTYEMVDePATDAVVaWtpSGt-SFVVlsqaDFcRdLLPKYFKHNNFSSFVRQLNTYGFRKVDPeqWEFANEeFIRdQRHrLKnIhRRK
TaHsfA4c   PAPFLiKTYEMVeDPATsrVVSWGpgGa-SFVVWnPpDFsRdLLPKYFKHNNFSSFiRQLNTYGFRKiDPeRWEFANddFIRGhmHLLKnIhRRK
TaHsfA4d   PAPFLiKTYEMVeDPATnrVVSWGpgGa-SFVVWnPpDFsRdLLPKYFKHNNFSSFiRQLNTYGFRKiDPeRWEFANddFIRGhmHLLKnIhRRK
TaHsfA4e   PAPFLiKTYEMVeDPATsrVVSWGpgGa-SFVVWnPpDFsRdLLPKYFKHNNFSSFiRQLNTYGFRKiDPelWEFANddFIRGhmHLLKnIhRRK
TaHsfA4f*                                              PQvlKHNNFSSFVRQLNTYGFRKVDPeqWEFANEeFLRdQRHrLKnIhRRK
TaHsfA5a   PAPFLlKTYEMVDDPATDAVVSWsdAsdTSFVVWnspEFAarLLPaYFKHsNFSSFiRQLNTYGFRKiDPeRWEFgNEyFvkGQKHLLKnIyRRK
TaHsfA5b   PAPFLlKTYEMVDDPsTDAVVSWsdAsdTSFVVWnspEFAarLLPaYFKHsNFSSFiRQLNTYGFRKiDPeRWEFgNEyFvkGQKHLLKnIyRRK
TaHsfA6a   PpPFLTKTfDlVaDPATDgVVSWGrAGN-SFVVWDPhlFAavLLPRfFKHsNFSSFVRQLNTYGFRKiDPDRWEFANEGFIRGQRqLLKmIkRRr
TaHsfA6b*                                                                 GFRKiDPDRWEFANEGFIRGQRqLLKmIkRRr
TaHsfA6c*                                                                 GFRKVDPDRWEFANEGFLRGQRHLLKtIkRRK
TaHsfA6d*                                                                 GFRKVDPDRWEFANEGFLRGQRHLLKmIkRRK
TaHsfA6e   PpPFLTKTYDlVeDPATDqVVSWGrAGN-tFVVWDPhvFAeaLLPRlFKHsNFSSFVRQLNTYGFRKVDPDRWEFANEGFLRGQRHLLKmIkRRK
TaHsfA6f*  PpPFLTKTfDlVaDPATDgVVSWGrAGN-SFVVWDPhlFAavLLPRfFKHsNFSSFVRQLNTY
TaHsfA7a   vAPFLaKTfDMVeDPATDAVVSWGaArN-SFVVWDPhaFAarLLPRhFKHaNFSSFlRQLNTYGFRKVnPDRWEFANtGFLgGQRHLLagIrRRr
TaHsfA7b   vpPFLaKTfDMVeDPATDAVVSWGaArN-SFVVWDPhaFAarLLPlhFKHaNFSSFlRQLNTYGFRKVnPDRWEFANtGFLgGQRHLLagIrRRr
TaHsfA8a   vAPFLTKvYDMVsDaATDkVmSWtdAGN-SFViWDahaFeRdLLsRhFKHrNFSSFiRQLNTYGFRKVDPDRWEwANEGFLRGQKHLLKiIkRkK
TaHsfA8b*                                                                 GFRKVDPDRWEwANEGFLRGQKHLLKiIkRkK
TaHsfA8c*                                                                 GFRKVDPDRWEwANEGFLRdQKHLLKiIkRkK
TaHsfB1a   PAPFLTKThqMVeergTDeViSWGeqGr-SFVVWkPvElARdLLPlhFKHcNFSSFVRQLNTYGFRKVvPDRWEFANEnFrRGeqgLLsgIrRRK
TaHsfB1b   PAPFLTKThqMVeergTDeViSWsehGr-SFVVWkPvElARdLLPlhFKHcNFSSFVRQLNTYGFRKVvPDRWEFANEnFrRGeqsLLsgIrRRK
TaHsfB1c   PAPFLTKThqMVeergTDeViSWsehGr-SFVVWkPvElARdLLPlhFKHcNFSSFVRQLNTYGFRKVvPDRWEFANEnFrRGeqsLLsgIrRRK
TaHsfB2a   PtPFLnKTYqlVDDPAvDdViSWsedGs-aFiVWrPaEFARdLLPKYFKHNNFSSFVRQLNTYGFRKivPDRWEFANdcFrRGeKrLLcdIhRRK
TaHsfB2b   PtPFLnKTYqlVDDPAvDdViSWsedGs-aFiVWrPaEFARdLLPKYFKHNNFSSFVRQLNTYGFRKivPDRWEFANdcFrRGeKrLLcdIhRRK
TaHsfB2c   PtPFLTKTYqlVeDPAvDdViSWGedGs-tFVVWrPaEFARdLLPKYFKHNNFSSFVRQLNTYGFRKivPDRWEFANdcFrRGeKrLLcdIhRRK
TaHsfB2d   PtPFLTKTYqlVeDPAvDdViSWGedGs-tFVVWrPaEFARdLLPKYFKHNNFSSFVRQLNTYGFRKivPDRWEFANdcFrRGeKrLLcdIhRRK
TaHsfB2e   PtPFLTKTYqlVeDPAvDdViSWGedGs-tFVVWrPaEFARdLLPKYFKHNNFSSFVRQLNTYGFRKivPDRWEFANdcFrRGeKrLLcdIhRRK
TaHsfB4a   PAPFLTKTYqlVDDPcTDhiVSWGedda-tFVVWrPpEFARdLLPnYFKHNNFSSFVRQLNTYGFRKivaDRWEFANEfFrkGaKHLLaeIhRRK
TaHsfB4b   PAPFLTKTYqlVDDPcTDhiVSWGedda-tFVVWrPpEFARdLLPnYFKHNNFSSFVRQLNTYGFRKivaDRWEFANEfFrkGaKHLLaeIhRRK
TaHsfB4c   PAPFLTKTYqlVDDPATDhiVSWGddrvstFVVWrPpEFARdiLPnYFKHNNFSSFVRQLNTYGFRKVvPeRWEFANEfFrkGeKqLLceIhRRK
TaHsfC1a   tAPFvaKTYqMVcDPrTDAlVrWGkgnN-SFlVtDvagFsQlLLPcfFKHgNFSSFVRQLNTYGFRKVhPDRWEFAhEsFLRGQtHLLprIvRRK
TaHsfC1b   vAPFvaKTfhMVsDPATDAVVcWGgAsN-tFlVlDPaaFsdfLLPsYFKHrNFaSFVRQLNTYGFRKVDPDvWEFAhEsFLRGQakLLplIvRkK
TaHsfC1c   vAPFvaKTfhMVsDPATDAVVcWGgAsN-tFlVlDPaaFsdfLLPsYFKHrNFgSFVRQLNTYGFRKVDPDvWEFAhEsFLRGQekLLplIvRkK
TaHsfC1d   vAPFvaKTfhMVsDPATDAVVcWGgAsN-tFlVlDPaaFsdfLLPsYFKHrNFaSFVRQLNTYGFRKVDPDvWEFAhEsFLRGQakLLplIvRkK
TaHsfC1e   tAPFvaKTYqMVcDPrTDAlVrWGkgnN-SFlVtDvagFsQlLLPcfFKHgNFSSFVRQLNTYGFRKVhPDRWEFAhEsFLRGQtHLLprIvRRK
TaHsfC2a   aAPFvwKTYrMVeDPgTDgVigWGkgnN-SFVVaDPfvFsQtmLPahFKHNNFSSFVRQLNTYGFRKVDPDRWEFAhgsFLRGQtHLLRnIvRRg
TaHsfC2b   aAPFvwKTYrMVeDPgTDgVigWGkgnN-SFVVaDPfvFsQtmLPahFKHNNFSSFVRQLNTYGFRKVDPDRWEFAhgsFLRGQtHLLRnIvRRg
TaHsfC2c   vAPFvaKTYgMVDDrATDAgVaWGsAGN-SFVVaDPfaFsemLLPahFKHsNFSSFVRQLNTYGFRKVDPDRWEFAhasFLRGQtHLLprIvRRq
TaHsfC2d   vAPFvaKTfaMVDDPATDAVVrWGpAsN-SFVVaDPftFshaLLPahFKHaNFSSFVRQLNTYGFRKVDPDRWEFAhasFLRGQtHLLprIvRRr
TaHsfC2e   vAPFvaKTYaMVDDPATDAVVaWGpAsN-SFVVaDPfaFsRaLLPahFKHaNFSSFVRQLNTYGFRKVDPDRWEFAhacFLRGQtHLLprIvRRr
TaHsfC2f   vAPFvaKTYaMVDDPATgAVVaWGpAsN-SFVVaDPfaFsemLLPahFKHsNFSSFVRQLNTsGFRKVDPDRWEFAhasFLRGQtHLLprIvRRs
TaHsfC2g   vAPFvaKTYaMVDDPATDAVVaWGpAsN-SFVVaDPfaFsRaLLPahFKHaNFSSFVRQLNTYGFRKVDPDRWEFAhacFLRGQtHLLprIvRRr
```

Supplementary Figure S2. Multiple sequence alignment of the DNA-binding domains of TaHsf proteins. Conserved amino residues are in upper-case letters and are highlighted. Gene with a partial DNA-binding domain is indicated with an asterisk.

```
               HR-A core               Insert                  HR-B
TaHsfA1a     LKRDKNVLMQELVRLRQQQQTTDHQLQTLGKRLHGMEQRQQQMMSFLAKAM
TaHsfA1b     LKRDKNVLMQELVRLRQQQQTTDHQLQTLGKRLHGMEQRQQQMMSFLAKAM
TaHsfA1c     LKRDKNVLMQELVRLRQQQQTTDHQLQTLGKRLHGMEQRQQQMMSFLAKAM
TaHsfA2a     LKRDRQLLMAEVVKLRQEQQNTRSDLQAMEKRLQGTEQKQQQMMSFLARVM
TaHsfA2b     LKRDKQLLMAEVVKLRQEQQNTKARLKAMEDRLRGTEQKQQQMTSFMARIL
TaHsfA2c     LKRDKQLLMAEVVKLRQEQQNMKVHLKAMEDRLRGTEQKQQQMTSFMARIL
TaHsfA2d     LKRDKQLLMAEVVKLRQEQQNTRSGLQAMEKRLQGTEQKQQQMMSFLARVM
TaHsfA2e     LKRDKQLLMAEVVKLRQEQQNTKARLKAMEDRLCGTEQKQQQMTSFMARIL
TaHsfA2f     LKRDKQLLMAEVVKLRQEQQNTRSDLQAMEKRLQGTEQKQQQMMSFLARVM
TaHsfA2h     LKRDKQLLMAEVVKLRQEQQNMKVHLKAMEDRLRGTEQKQQQMTSFMARIL
TaHsfA3a     LRREKNALLQEVARLKEEHNQTIEHMNALNQRLETAEDRQKQVVSFLAKLL
TaHsfA4a     LKCDNASLKLQLERKKTD---MESKMKVLEDKLFAIEGQQKNLISYVREIV
TaHsfA4b     LKCDNASLKLQLERKKTD---MESKMKALEDKLFAIEDQQKNLISYVREIV
TaHsfA4c     LKHENSVLVAELQRQAQQQCGLSWLMQSLEERLLAMEQRQADVVASVRDIQ
TaHsfA4d     LKHENSVLVAELQRQARQQCGLSWLMQSLEDRLMAMERRQADVVASVRDTL
TaHsfA4e     LKHENGLLVAELQRQAQQQCGLSWLMQSLEDRLTAMEQRQANVVASVRDTL
TaHsfA4f     LKCDNASLNLQLERKKTD---MESKMKALEDKLFAIEDQQKNLISYVREIV
TaHsfA5a     LAREKANLQAELWKSKQQESGTMFQIEALERRAVDMEQRQGKMIAFLQQAS
TaHsfA5b     LAREKANLQAELWKSKQQESGTMFQIEALERRAVDMEQRQGKMIAFLQQAS
TaHsfA6a     LKRDKNALLEEVVKLRQEQQSSRADMRAMEERLHRVEQKQLQMMGFLARAI
TaHsfA6b     LKRDKNALLAEVVKLRQEQQSSRADMRAMEERLHHVEQKQLQMMGFLARAI
TaHsfA6c     LKRDKNLLITEVVKLRQEQQATKDNVQAMEGRLRAAEQRQAQMMGFLARAM
TaHsfA6d     LKRDKNLLITEVVKLRQEQQATKDNVQAMEGRLRAAEQRQAQMMGFLARAM
TaHsfA6e     LKRDKNLLITEVVKLRQEQQATKDNVQAMEGRLRAAEQRQAQMMGFLARAM
TaHsfA7a     LRRDREALKRELAGLKRQQEEARATLLDMERRVQGTERRQEQCKAFLARAV
TaHsfA7b     LRRDREALKRELAGLKRQQEEARATLLDMERRVEGTERRQEQCKAFLARAV
TaHsfA8a     LKRDKSLLMQQLVDLRHYQQSSNLEVQSLIQRLQVMEQNQKQMMALLSIVV
TaHsfA8b     LKRDKALLMKQLVDLRLYQQSSNLEVQSLIQRLQVMEQNQKQMMALLAIVV
TaHsfA8c     LKRDKALLMKQLVDLRLYQQSSNLEVQSLIQRLQVMEQNQKQMMALLAIVV
TaHsfB1a     LRKDNHALAAELALARRHC---------------------EELLGFLSRFL
TaHsfB1b     LRKDNHALAAELALARRHC---------------------EELLGFLSRFL
TaHsfB1c     LRKDNHALAAELALARRHC---------------------EELLGFLSRFL
TaHsfB2a     LRRENSRLTRELGQMKKLC---------------------NNIFVLMSKYT
TaHsfB2b     LRRENSRLTRELGQMKKLC---------------------NNIFVLMSKYT
TaHsfB2c     LRRDNARLTRELGQMKKLC---------------------NNIVSLMSKFA
TaHsfB2d     LRRDNARLTRELGQMKKLC---------------------NNIVSLMSKFA
TaHsfB2e     LRRDNARLTRELGQMKKLC---------------------NNIVSLMSKFA
TaHsfB4a     LRRRNSLLLSELAHMKKLY---------------------NDIIYFLQNHV
TaHsfB4b*    LRRHNSLLLSELAH
TaHsfC1a     LRQEQTAIGEQLAKMSRRLQA--------------TERRPDRLMSFLSKLA
TaHsfC1b     LRDERRGMEEELQDMDRRLCA--------------AENRPGQMMAFLGKLA
TaHsfC1c     LRDERRGMEEELQAMDRRLCA--------------AENRPGQMMAFLGKLA
TaHsfC1d     LRDERRGMEEELQAMDRRLRA--------------AENRPGQMMAFLGKLA
TaHsfC1e     LRQEQTAIGEQLAQMSRRLQA--------------TERRPDRLMSFLSRLA
TaHsfC2a     LKKEQSTIDDRVAAMWRRVQE--------------TERKPKQMLAFLLTIV
TaHsfC2b     LKKEQNTIDDRVAAMWRRVQE--------------TERKPKQMLAFLLTVV
TaHsfC2c     LRREQRATEERVAAMWRRVQE--------------TERRPKQMLGFLLKVV
TaHsfC2d     LKEEQRATEDRLASMRRRVQD--------------AERRPKLMLAFLLKVV
TaHsfC2e     LKEEQRATEDRLAAMWRRVQD--------------AERRPKLMLAFLLKVV
TaHsfC2f     LRNEQRATEESMAAMWRRVQD--------------AERRPKLMLAFLLKVV
TaHsfC2g     LKEEQRATKDRLAAMWRRVQD--------------AERRPKLMLAFLLKVV
```

Supplementary Figure S3. Multiple sequence alignment of HR-A core and HR-B regions of TaHsf proteins. Conversed hydrophobic positions of HR-A core and HR-B are highlighted. Gene with a partial HR region is indicated with an asterisk.

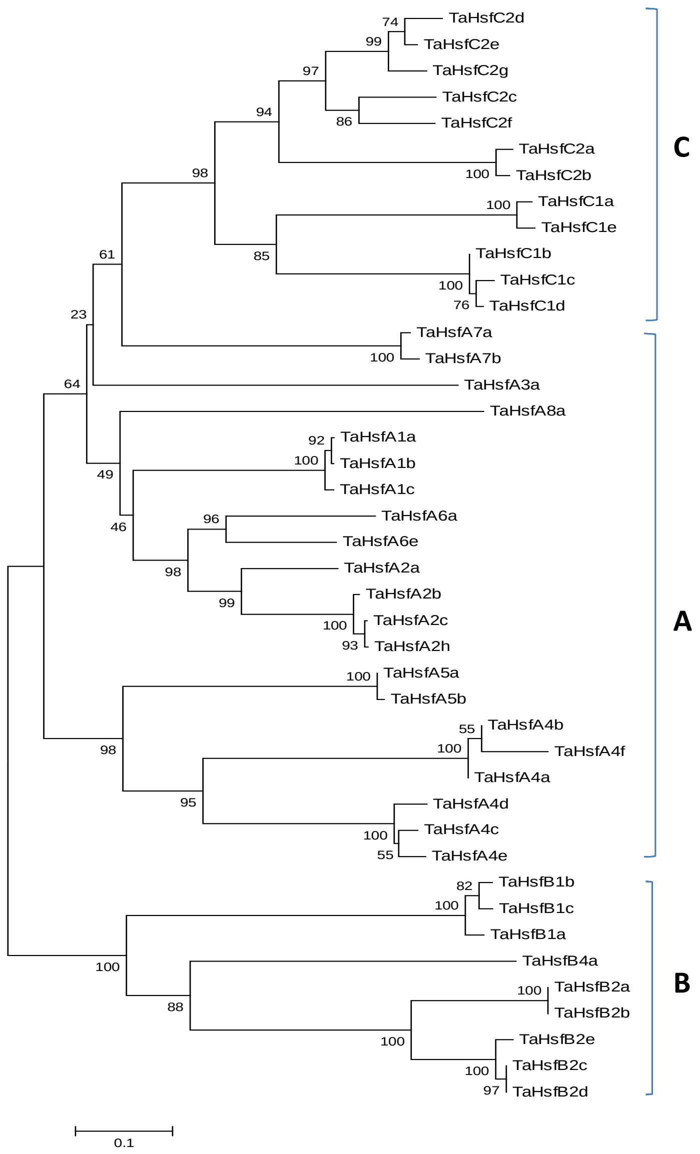

Supplementary Figure S4. Neighbor-joining phylogenetic tree of wheat Hsf proteins

The N-proximal regions (from the start of DNA-binding domain to the end of HR-A/B region) of Hsf proteins were used for construction of the phylogenetic tree using MEGA 5.10 program.

TaHsf proteins that contain partial DNA-binding domain and HR-A/B sequences are excluded from this analysis. Un-rooted neighbor-joining analysis was performed with pairwise deletion and Possion correction. Bootstrap values are indicated.

Three classes of TaHsf proteins are indicated. It appears that the subclass A7 members are more closely related to the class C member in this tree.

TaHsp26.6 promoter
AGGAAGTGGTAGATGGTAGATGTACAATGATAACATTTATATTACTAAAAAGGAAATATTAATTTATGTCATTATAAATATTCATAC
ATATAAAGCAATGTTAATCATATTTTTAAAGATATACACATATAGTTATATTTGTGTATTTTTAGAAGTTCAAATATATTTTTAAAT
GTTCACACTGATGAAATAAAATATCCATCATGTATATAACAACTATTCCTACATAATAGTATAATACCTTATGCTTATCTATTTATT
TTTAAAAGTTCATATATTTTAAGAAGTATACATACACATCTGAATATTTTTGGAGGATATAAATATATGTTCATACAACGTGGAAAA
ATGTTGGCATCCAAAGAGGACTAAAATATAAAAAAGTCTAAGATGATTTGGATATTTCATTATGGAATATATATAGACTTTAATGAG
TGAACTAACACACTAAAAGGTCAACATTACGAGATGGGCCTCGGCTTCACGGTCTGAAATAAATTTTCATGTTGGGCCTCCTGATCG
CGAAGTGCCGAGAAAGTGTTCGAGAATTATCGAGAGAACCCCACAAACCTGAAGAACACCCGGGCCTCTCCAACCAGGTACTCTGAG
CGGTGTGCGTTGTCCCGAAGCTTCAACATCTTTCCCCATCGTTCCACGATCATGGCATGAAATCCCCACCGCTCTATAAAAGGGCGC
GGCAAAGTCGCCATCTCCCACCGAACAAGCGAGCAAGATAAGCATTGGCTTCCGAGTTCCAACGATCTCGTAGACACTCTCTCGTTT
CAATTCTCGACCGACATTTAATCTTCCAAAGCTCCGTATCTGGTGCA**ATG**GCCGCAGCGAATGCCCCCT

TaHSP70d promoter
GGTGAACCCGTCCGTACTCCGCCAGAATCACTGCATCTCCGCGGAAAGCCCACGGTCCATCCTCCATCACGCGCTCCCAATCACCGA
GGCAAGAAAACTGCAGAGTGTATAAATTGTCCTCGAGTGGTCGGATGTTCACCTTCTGCGCCAGATCCCAAGCGGCCCTCATGTTCT
TGTAGAACCAGAATTGGCTATAGGGCTTTTCATTGTAAACTCTAGCAATCGCCATCCACCGCGTTGCCTCCGGCGGTACAACTTTTT
GATCAACAACAACGTCATCTAGGTCCTCCTCCGAGAGACCCAATTCCTTCATCATCGCCTCCAGATCCTCGTGAGTGCCCGATCCAG
AAGCTTCGCTCGCCATGTTCGTTCCCTAGCCCGAAGAACTCTCCACCGGGCTTGTTTGTCGAAACCCTAGACTCCCTTCCTCTCCAC
AGACCCCTAACCAAGATCGGGTAGCAGTCCAGGGACCGCCCCTTGATCGATCTGAGTCGAGGGATCACGGAGAATCTAGGGTAGGGA
AGACGAATCTCTACGGCGGCTGGGAGTCGCCACCGAGAGGAGAGGAACCCTAACGTGAGGGAAAAAGACTTCCAGAGCCCGGAAGCT
ATCATTCAAAAAAAAAAGGCACGTACTAACATACGTGGGCCGAGACTAGGCCTGGCCCAATTACCCGTGCAGGCCGAGGCAGGCTTGG
CTAGGACGATTGATGTTATTCTAAAAAAAACGATTGATGTAAAAAAAAGAACTACTAGAATCCTAGGTTTTTCCTTCCGCGCGTCCT
ACCCACCCGACGCGTGCTCCTACCACCGTCTCTCCACAGTAGCCTACCATGCTCGTGCACATTGTAGAACCTACCAGCACCACCAGA
AGCGCCCGCGCGTGGGCCACCCCACCCCACCCCACCGCCACACCGCCCCGCCCCGCACACTCCGGACCCTTCCAGAACCATCACCCC
CTCCCCGATCCGACGGCCAGGATCGCACGCGCCAGAACATTCCAGACCCGGCCGCCCGCCGCCTCCGCCATAAAACCCCGCCCGTCT
CCGGCCCTCCCTCCCCACTTCCGTTCGAAATCGAAATCAGAGAGGGGCAAAGCAAATCGCACCAGGCAAACTCAGAGGGTCTTCCGG
CGAACCCCAAAGCGAGAGAGCGAGCGAGCGATTCCCAGGAGAGGAGAGGAGGCGGAG**ATG**GCCAAGGGC

Supplementary Figure S5. *TaHsp26.6* and *TaHsp70d* promoter sequences assembled from sequence databases.

TaHsp26.6 promoter was assembled from the wheat genome sequences at http://www.cerealsdb.uk.net/CerealsDB/Documents/DOC_search_reads.php (IDs**:** F2S221K01D4GPV, GHY43EF02DOPA2, F676MBK02G2HTR, GKY3Q6202J1EVR, GGWFHOC02HN4ZY) and *TaHsp26.6* cDNA (AF097659).

TaHSP70d promoter was assembled from the wheat genome sequences (IDs: GCMIFJO01CTPEJ, GIDSL3P07GHOXX, GKTGI0Q01BPTO4, GHY6XPR07JAKCJ, F01XQWW01C8F0P, GCRXJAC01AD6J) and *TaHsp70d* cDNA (AF005993).

The translation start codon ATG is in bold.
